# Long-range proton and hydroxide ion transfer dynamics at the water/CeO_2_ interface in the nanosecond regime: reactive molecular dynamics simulations and kinetic analysis†

**DOI:** 10.1039/d4sc01422g

**Published:** 2024-04-02

**Authors:** Taro Kobayashi, Tatsushi Ikeda, Akira Nakayama

**Affiliations:** a Department of Chemical System Engineering, The University of Tokyo Tokyo 113-8656 Japan t_ikeda@chemsys.t.u-tokyo.ac.jp nakayama@chemsys.t.u-tokyo.ac.jp

## Abstract

The structural properties, dynamical behaviors, and ion transport phenomena at the interface between water and cerium oxide are investigated by reactive molecular dynamics (MD) simulations employing neural network potentials (NNPs). The NNPs are trained to reproduce density functional theory (DFT) results, and DFT-based MD (DFT-MD) simulations with enhanced sampling techniques and refinement schemes are employed to efficiently and systematically acquire training data that include diverse hydrogen-bonding configurations caused by proton hopping events. The water interfaces with two low-index surfaces of (111) and (110) are explored with these NNPs, and the structure and long-range proton and hydroxide ion transfer dynamics are examined with unprecedented system sizes and long simulation times. Various types of proton hopping events at the interface are categorized and analyzed in detail. Furthermore, in order to decipher the proton and hydroxide ion transport phenomena along the surface, a counting analysis based on the semi-Markov process is formulated and applied to the MD trajectories to obtain reaction rates by considering the transport as stochastic jump processes. Through this model, the coupling between hopping events, vibrational motions, and hydrogen bond networks at the interface are quantitatively examined, and the high activity and ion transport phenomena at the water/CeO_2_ interface are unequivocally revealed in the nanosecond regime.

## Abstract 2

The structural properties, dynamical behaviors, and ion transport phenomena at the water/CeO_2_ interface are investigated by reactive MD simulations employing NNPs, and a counting analysis based on the semi-Markov process is formulated and performed.

## Graphical abstract

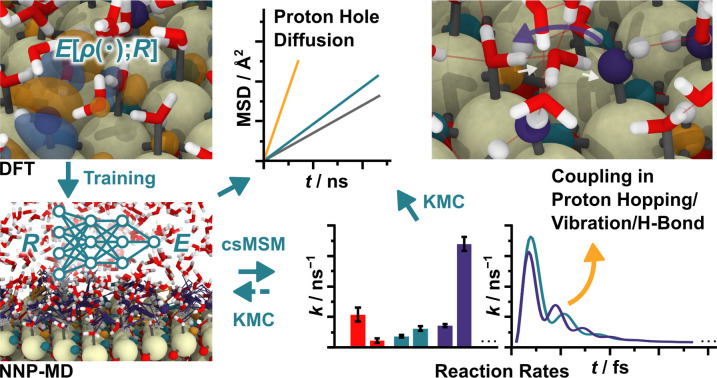

## 1. Introduction

A comprehensive understanding of the interface between water and metal oxides is fundamental due to its significance in a wide range of physicochemical phenomena and technological applications, such as catalysis, polishing, biomineral formation, colloid chemistry, and corrosion (refer to ref. 1 and 2 for examples). The behavior of molecules at the water/metal oxide interface is generally complex, and probing the microscopic nature of the interface remains a formidable challenge in experiments. Molecular dynamics (MD) simulations provide a bottom-up picture of water/metal oxide interfaces and have proven to be invaluable tools for microscopic understanding of the structural properties and dynamical behaviors of water molecules at the interface. While MD simulations have been successfully applied to various types of water/metal oxide interfaces, their reliability is contingent on the potential used in the simulation. In particular, metal oxide surfaces are generally reactive, and solvent molecules dissociatively adsorb through the acid–base sites on the surface; therefore, it is not straightforward to apply empirical force fields to the interfaces. In this respect, the development of empirical force fields that can describe bond breaking/formation and chemical reactions is in high demand. The reactive force field (ReaxFF) is one of the most successful reactive force fields^3,4^ and has been applied to various types of interfaces, including water/cerium oxide (CeO_2_) interfaces,^5^ allowing for large-scale and long-time simulations. Its accuracy is, however, limited by the complex functional form of the force field.

DFT-based molecular dynamics (DFT-MD) simulations, also referred to as first-principles MD or *ab initio* MD, have been widely used to investigate liquid/metal oxide interfaces owing to rapid advances in computational power.^6^ DFT calculations are performed at each timestep of the MD simulations, making this method applicable to complex systems that involve chemical bond breaking/formation with a high degree of accuracy. DFT-MD simulations have been successfully applied to various liquid/metal oxide interfaces, and in particular, for water interfaces, simulations for TiO_2_,^7–11^ CeO_2_,^12–15^ Al_2_O_3_,^16–20^ ZnO,^21,22^ ZrO_2_,^23^*etc.* have been reported, and the interface structures and hydrogen bond networks, including the proton hopping mechanism, have been scrutinized in these studies.

Currently, DFT-MD simulations can be executed for systems containing several hundred atoms over simulation times of several hundred picoseconds. At liquid/metal oxide interfaces, proton hopping is generally active, and these events typically occur several times or less in a picosecond per site. It is, therefore, sometimes difficult to obtain statistically converged results with respect to proton hopping and dissociation equilibrium within the timescale allowed by DFT-MD simulations. Thus, it is highly desirable to establish simulation methods that can treat bond breaking/formation at the liquid/metal oxide interface with reliable accuracy for large-scale and long-time simulations. DFTB (tight-binding),^24^ the divide-and-conquer method,^25^ and the fragment molecular orbital method^26^ are those of such successful applications, where in the latter two cases, the reliability of these methods depends on the sensible partitioning of the system.

In recent years, neural network potentials (NNPs)^27–29^ have attracted considerable attention for providing a reliable description of interatomic interactions for various types of bonding with high fidelity. NNPs possess functional forms to predict potential energies and forces at given configurations and usually use training data obtained by electronic structure calculations such as DFT. NNPs include higher-order many-body effects and can treat bond breaking/formation and, thus, chemical reactions. In principle, a properly trained NNP guarantees the accuracy of the underlying data, and the computational costs are significantly lower than those in direct DFT-MD simulations, making it possible to perform large-scale and long-time simulations. Several successful applications using NNPs have been reported at the interface of water/metal oxide; they include simulations of interfaces with ZnO,^30–32^ TiO_2_,^33–35^ and Fe_2_O_3_.^36^

This study aims to construct NNPs for investigating the interface of water and CeO_2_ with the two low-index surfaces of (111) and (110) and to gain insight into the structural properties and dynamical behaviors of water molecules at interfaces by long-time and large-scale simulations that are inaccessible by direct DFT-MD simulations. CeO_2_ has received considerable attention due to its immense scientific and technological importance in the fields such as catalysis, glass polishing, biomedical technology, and sensors (see ref. 37–42 for recent reviews). Several DFT-MD simulations have been performed for the water/CeO_2_ interface,^12–14^ and high activity involving proton hopping has been reported. The high activity, however, makes it difficult to construct robust NNPs since the training datasets should properly include data on various proton hopping patterns with diverse configurations of surface adsorbates and hydrogen-bond networks. Here, to accumulate training data, we use the DFT-MD method in conjunction with enhanced sampling methods and a refinement scheme to cover low-probability structural configurations, which is essential for running stable and reliable NNP-based MD simulations (NNP-MD). After successfully training NNPs, we run NNP-MD simulations on the nanosecond timescale and investigate the intricate dynamics of water dissociation and the proton hopping mechanism at the water/CeO_2_ interface. Furthermore, we formulate and perform a kinetic analysis based on a semi-Markov process to extract the reaction rates of proton/hydroxide ion transfers at the interfaces from the NNP-MD trajectories. The coupling between hopping events, vibrational motions, and hydrogen bonding lifetimes is quantitatively discussed by the time-dependent analysis of the reaction rates.

## 2. Computational details

### 2.1 DFT calculations for a slab model of CeO_2_ surfaces

All DFT calculations in this study were conducted using periodic boundary conditions, employing the mixed Gaussian and plane waves (GPW) approach, as implemented in the CP2K program package.^43^ The short range variants of the double-ζ valence plus polarization (DZVP) basis sets of the MOLOPT type^44^ were employed for H, C, N, and O atoms to represent the valence electrons, and the norm-conserving Goedecker–Teter–Hutter pseudopotentials^45,46^ were used to describe the interactions between the valence and core electrons. For Ce atoms, we employed the basis sets and pseudopotential generated by Wang and coworkers.^47^ The energy cutoff of 800 Ry was used for the auxiliary plane wave expansion of the density. The generalized-gradient approximation (GGA) with Perdew–Burke–Ernzerhof (PBE) functional models^48^ was employed as the exchange and correlation potential, and the DFT+U approach was taken in order to represent the nature of 4f orbitals of Ce atoms correctly.^49,50^ The *U* value was set to 7.0 eV following previous work.^47^ The DFT-D3 (zero damping) method was used to incorporate dispersion forces.^51^ The Brillouin zone integration was performed with a reciprocal space mesh consisting of only the *Γ*-point in the GPW approach. The convergence criteria for the energy in the SCF calculation were set to 1 × 10^−6^ hartree.

CeO_2_ has a fluorite structure in the low-temperature regime. The lattice parameters of bulk CeO_2_ were determined by cell optimization of the 3 × 3 × 3 supercell of the conventional structure, and the optimized lattice constant of 5.427 Å was in good agreement with the experimental value of 5.411 Å,^52,53^ where the deviation from the experimental value was less than 1%. The low-index facets of (111) and (110) were considered in this study. It has been determined theoretically^54^ and experimentally^55^ that the (111) surface is the most stable low-index surface among the single crystal terminations of CeO_2_ and represents the majority of the surface area of CeO_2_ nanoparticles. The surface energy is in the order of (111) < (110) < (100),^13,50,56^ where the surface energies were reported to be 0.81, 1.26, and 1.51 J m^−2^, respectively.^13^

**Fig. 1:**
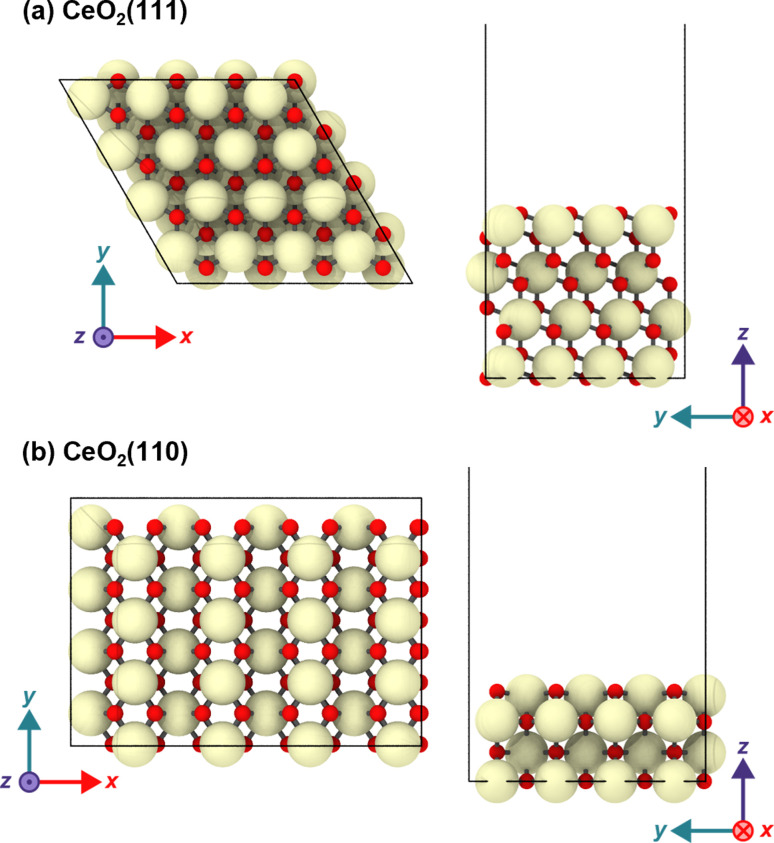
Top and side views of the (a) CeO_2_(111) and (b) CeO_2_(110) slabs.

The CeO_2_(111) surface was modeled as a periodic *p*(4 × 4) hexagonal slab of 64 CeO_2_ units with four O–Ce–O tri-layers (see Fig. 1). The dimensions of the simulation cell were set to *a* = *b* = 15.35, *c* ∼ 56 Å, *α* = *β* = 90°, and *γ* = 60°, and this slab was separated by a vacuum space of ∼38 Å in the direction perpendicular to the surface. The CeO_2_(110) surface was modeled with a *p*(4 × 4) slab with four atomic layers, and the size of the simulation cell was set to *a* = 21.71, *b* = 15.35, *c* ∼ 51 Å and *α* = *β* = *γ* = 90°, and the slab has a vacuum space of ∼38 Å. In both models, the bottom O–Ce–O tri-layers were fixed at the bulk positions during the geometry optimization and MD simulations. The structure visualization in the figure was carried out using OVITO.^57^

### 2.2 DFT-based molecular dynamics (DFT-MD) simulations

The quality and applicability of NNPs strongly depend on the training data, and it is natural to use the energies and forces from the DFT-MD trajectory as the training data. However, when the standard canonical ensemble (NVT) DFT-MD method is used, it is likely that the configurations close to the bond breaking/formation events are not sufficiently included. Biased sampling or enhanced sampling simulations are preferable to efficiently include these configurations. Some of the recent studies employed enhanced sampling methods such as metadynamics for exploring the configuration space to accumulate the training data.^58–61^

For the simulation of the water/CeO_2_ interface, simulation cells were prepared with 191 and 331 water molecules placed on the (111) and (110) surfaces, respectively. The vacuum space was placed between the water layer and the repeated slab above, which had a height of approximately 15 Å. The mass of hydrogen was replaced by that of deuterium, allowing for a larger timestep of 1.0 fs. For each simulation cell, the system was first equilibrated at 360 K under NVT conditions with Nosé–Hoover thermostats. Previous studies have shown that increasing effective simulation temperature is a good compromise for describing the structural dynamics of actual ambient liquid water due to the strong water–water interactions in PBE and its derived functionals.^62–64^ For each interface, a total of 25 ps simulation was performed, and trajectories in the last 20 ps were used as NNP training data.

In order to increase the diversity in the configuration in the dataset, a total of 20 ps simulations were performed with enhanced sampling techniques to accelerate the reactive events more frequently by using biased MD (BMD) and temperature-accelerated MD (TAMD)^65,66^ and these trajectory data were added into the training dataset. In short, proton hopping between water molecules and the surface oxygen atoms was enhanced by BMD, in which the bias potential to flatten the energy barrier of the proton hopping was added to the system potential. TAMD was introduced to promote proton hopping of water molecules or hydroxide ions coordinated to surface Ce atoms. The PLUMED program^67,68^ was employed to perform the enhanced sampling simulations. In order to retrieve pure DFT forces without artificial forces originating from enhanced sampling techniques and those imposed by slab constraints, in-house modifications were applied to the CPK2 program. The simulation details of BMD and TAMD are given in the ESI.†

### 2.3 Neural network potential (NNP) based molecular dynamics (NNP-MD) simulations

In this work, the DeepPot-SE descriptor model^69^ was adopted, and the NNPs were constructed using the DeePMD-kit.^70–72^ In this model, the potential energy of an entire system is represented as the sum of all atomic energies. The potential energy of the system is then represented as1
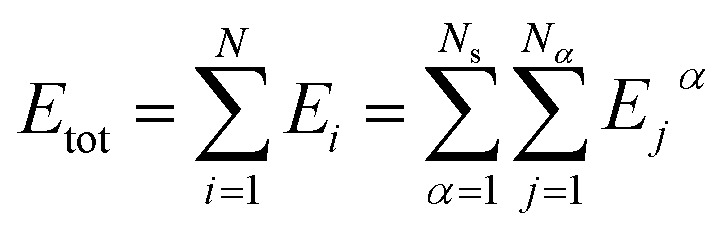
where *N* is the total number of atoms in the system, *N*_s_ represents the number of atomic species, and *N*_*α*_ is the number of atoms belonging to atomic species *α*. The local energy *E*_*j*_^*α*^ of each atomic species depends on the positions of the surrounding atomic species within a cutoff radius *R*_C_ through the so-called symmetric functions, which are expressed using deep neural networks (DNNs) in the DeepPot-SE descriptor model. This functional form possesses symmetry with respect to translation, rotation, and exchange of the positions of atoms belonging to the same atomic species. Because the potential energy of the entire system is represented as a sum of the local energies, the NNP can be used for systems with different numbers of atoms or sizes of simulation cells from which the training data are generated.

In this study, the DNNs were trained by the iterative procedure with random initialization of the parameters. For each of the CeO_2_(111) and CeO_2_(110) surfaces, the DFT energies and forces of 4001 configurations with a 5 fs interval were taken from the standard NVT DFT-MD simulations of 20 ps, along with the 4001 configurations with the same interval of DFT-MD simulations with the enhanced sampling scheme where both TAMD and BMD were simultaneously applied. The cutoff radius *R*_C_ and smooth cutoff parameters *R*_SC_ were set to *R*_C_ = 7.5 Å and *R*_SC_ = 1.0 Å, respectively. Using these configurations as the training datasets, “preliminary” NNPs were trained.

The refinement of “preliminary” NNPs was carried out by using an idea similar to that implemented in the DP-GEN package.^73^ Four different NNPs were constructed using the same training data with different random initializations of the NN parameters, and then four independent NVT MD simulations with NNPs (referred to as NNP-MD hereafter) at 360 K were performed for 1 ns, each using a different NNP. LAMMPS^74^ was used for running NNP-MD simulations. The variances in the atomic forces predicted by the four NNPs were computed at intervals of 100 fs along each NNP-MD trajectory. The variance was used as a measure of the reliability of the NNPs, and a total of 250 configurations were selected from each trajectory, starting from the one with the largest value of the maximum variance of atomic forces. A total of 1000 configurations were then collected and single-point DFT calculations were performed on these configurations. The resulting energy and forces were added to the training data. Here, the configurations for which the single-point DFT calculations did not converge were excluded. The main difference between this procedure and DP-GEN is the simulation time of NNP-MD simulations per refinement step. We performed the NNP-MD simulations for 1 ns to efficiently collect slowly changing configurations that involve the rearrangement of the hydrogen bond network. Note that it was essential to include the configurations with the enhanced sampling scheme in the initial “preliminary” training dataset to initiate this refinement procedure because the NNP-MD using the NNP trained only with standard NVT DFT-MD failed in a short time in our system.

By repeating this refinement three times, more than 99% of the configurations in the last NNP-MD run were within 0.2 eV Å^−1^ for the maximum value of variances of atomic force components, which was considered to be converged. The details are given in the ESI,† where the density profiles using the four NNP models and the maximum values of variances of atomic forces are provided. A total of 10 986 and 10 993 configurations were used to construct NNPs for the water/CeO_2_(111) and water/CeO_2_(110) interfaces, respectively. The root mean square errors (RMSEs) of the force components were evaluated using random 200 configurations from the last NNP-MD run that were not used in training. The atomic force RMSEs were estimated as 71.9 and 66.5 meV Å^−1^ for the water/CeO_2_(111) and (110) interfaces, respectively. Compared with the atomic force RMSEs reported in previous studies for water/solid oxide interfaces, 143.4 meV Å^−1^ for water/ZnO,^31^ 149.8 meV Å^−1^ for water/hematite,^36^ and 102.3 meV Å^−1^ for water/TiO_2_,^11^ the constructed NNPs are considered to achieve sufficient accuracy.

## 3. Results and discussion

### 3.1 Adsorption of a water molecule on the CeO_2_ surface determined by DFT calculations

In this subsection, the results of DFT calculations on the adsorption structure and energy of a single water molecule on the CeO_2_ surface are provided for reference in the discussion given later. The CeO_2_ surface has a strong Lewis base and a relatively weak Lewis acid site, and it has been experimentally known that water molecules are partially dissociated on the CeO_2_ surface.^75–77^ The adsorption structure of a water molecule has also been well-studied theoretically, and a water molecule can be molecularly or dissociatively adsorbed on the surface. It is generally accepted that the dissociative state is more stable than the molecular one on the two low-index surfaces of (111) and (110). The adsorption energies of a water molecule on the (111) and (110) surfaces were calculated in our simulation setup, and the details are given in the ESI.† On the CeO_2_(111) surface, the adsorption energy of the dissociative state is −0.75 eV, and it is slightly higher than that of the molecular state (−0.74 eV). These values are in good agreement with previous theoretical studies, where the adsorption energies range from −0.3 to −0.8 eV depending on the DFT functionals, slab sizes, surface coverage of water layers, *etc.* Theoretical studies on the adsorption of water molecules on CeO_2_(111) have been compiled in ref. 78. On the CeO_2_(110) surface, the adsorption energies of the molecular and dissociative states were calculated to be −1.06 and −1.24 eV, respectively, which are also in good agreement with other theoretical studies (see the ESI†).

### 3.2 Density profiles of oxygen and hydrogen atoms at the interface from NNP-MD simulations

Large-scale and long-time MD simulations were performed using NNPs to analyze the static and dynamic properties of the water/CeO_2_ interface. In order to obtain good statistics, the surface areas were increased from the DFT-MD simulation setup. The surface areas of *p*(8 × 8), *i.e.*, doubled in both *x*- and *y*-directions, were employed for both the CeO_2_(111) and (110) surfaces, while the slab thickness was the same as that in the DFT-MD simulations. The numbers of water molecules were 717 and 1153 for the CeO_2_(111) and (110) interfaces, respectively, which provide a water layer thickness of ∼20 Å. In running NNP-MD simulations, the hydrogen mass was restored to its original value, and the timestep was set to 0.5 fs. The Nosé–Hoover thermostats were attached to the system, and the temperature was controlled at 360 K. The NNP-MD simulations were performed for 5 ns, and the time course of the numbers of various surface species is shown in the ESI,† where the dissociation equilibrium is checked. After equilibrium was reached, the structures sampled every 5 fs from the last 4 ns were used for analysis. Snapshots of the two systems are given in ESI S.V.†

**Fig. 2:**
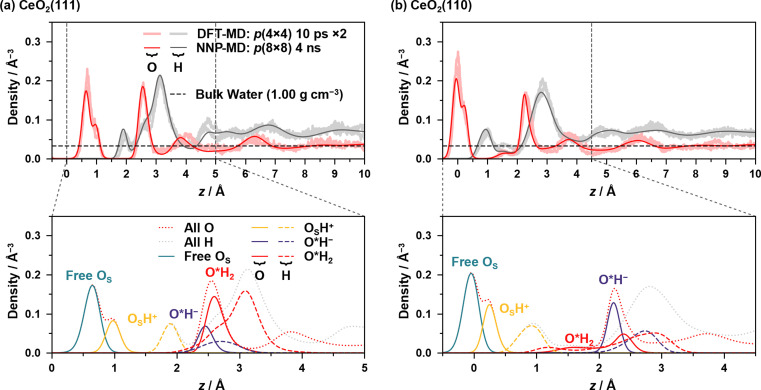
Density profiles of oxygen and hydrogen atoms at the (a) water/CeO_2_(111) and (b) water/CeO_2_(110) interfaces. The upper and lower panels show total and decomposed profiles, respectively. For comparison, the results from two independent 10 ps equilibrium trajectories of the NVT DFT-MD simulations are also included. Note that the mass of hydrogen was replaced by that of deuterium in the DFT-MD simulations. The density of bulk water is also shown in the upper panels.

Fig. 2 shows the density profiles of oxygen and hydrogen atoms along the *z*-axis, which is perpendicular to the surface, along with the decomposed profiles, where *z* = 0 is set to the average of *z*-coordinates of the outermost surface Ce atoms. Comparisons of the profiles with those of the conventional DFT-MD simulations are also shown, where two independent DFT-MD simulations were performed for 10 ps starting from different initial conditions. The apparent discrepancy between the two DFT-MD simulations indicates that the 10 ps of the DFT-MD simulation is insufficient and that simulations with a longer time are required.

In the following, O_S_ represents the surface O atoms, and hydrogen atoms are considered to be covalently bonded to their nearest O atom, which is also used in the proton hopping analysis below (hereafter referred to as the nearest-neighbor bonding (NNB) assignment). The dissociation of a water molecule on the surface leads to a surface hydroxy group (Ce–O_S_H^+^) and solvated hydroxide ion (Ce–O*H^−^) that resides on the surface Ce atom, and if only one H atom is assigned to a surface/water O atom by the NNB assignment, it is regarded as a surface hydroxy group or hydroxide ion. The O atoms of adsorbed water or hydroxide ions are designated with an asterisk, such as O*H_2_ or O*H^−^, indicating that the distance between the surface Ce and O atoms is less than 3.5 Å and 3.1 Å for the water/CeO_2_(111) and water/CeO_2_(110) interfaces, respectively. The appropriateness of these values was confirmed by the radial distribution functions of Ce–O (see ESI S.VI†).

Fig. 2(a) shows the density profiles at the water/CeO_2_(111) interface, and the prominent peak of O density at ∼0.7 Å represents the surface O_S_ atoms that are not surface hydroxy groups (O_S_H^+^), and a small peak at ∼1.0 Å represents the oxygen atoms of surface hydroxy groups. The integration of the O_S_H^+^ component yields an equilibrium fraction of surface hydroxy groups, showing that 29.8% of surface O atoms are hydroxylated (degree of hydroxylation). This is in good agreement with the previous work of DFT-MD simulations without dispersion correction, where 20–33% of the surface O sites are hydroxylated.^12,13^

The pronounced peak of O density at ∼2.6 Å represents the strong adsorption of water molecules (O*H_2_) or hydroxide ions (O*H^−^) to the surface Ce atoms, and it is seen that most of the surface Ce atoms are coordinated to water molecules or hydroxide ions, indicating highly structured coordination of water molecules or hydroxide ions. Integrations of the O densities of O*H_2_ and O*H^−^ show that 70.5% and 26.1% of the Ce sites are coordinated by water molecules and hydroxide ions, respectively, which means that 27.0% of adlayer water molecules are dissociated at the interface. These numbers also correspond to a water coverage of 7.57 H_2_O nm^−2^, including the hydroxide ions, and it is in good agreement with 7.33 H_2_O nm^−2^ in previous work.^13^ As indicated by the peak locations, O*H^−^ ions are more strongly bound to the Ce atom than O*H_2_ due to the strong electrostatic interactions. The integration of the peaks of O_S_H^+^ and O*H^−^ yields very similar values, the latter being slightly smaller.

For the density profiles of the H atoms on the CeO_2_(111) surface, a small peak at ∼1.9 Å corresponds to H atoms that are bound to surface O atoms as surface hydroxy groups. The broad peak from *z* = 2.2 Å to 4.0 Å represents the H atoms derived from O*H_2_, O*H^−^, and water molecules that are hydrogen-bonded to the surface O_S_ atoms without coordinating to the surface Ce atoms (see Fig. S7(c)–(i)† for detailed decomposed density profiles). The O*H_2_ component has a similar shape to the overall peak and determines the shape of the peak, where one of the OH bonds is parallel to the surface, and the other OH bond is oriented perpendicular to the surface. The density profile of the H atoms of water molecules that are not coordinated to the Ce atom exhibits two peaks, where the first peak corresponds to the H atoms forming a hydrogen bond to the surface O atom.

Fig. 2(b) shows the density profiles at the water/CeO_2_(110) interface, where two prominent peaks of O density at ∼0.0 Å and ∼2.2 Å are observed. The first peak corresponds to the surface O_S_ atoms, and the second peak represents the strong adsorption of O*H_2_ or O*H^−^. For the first peak, a small peak at ∼0.25 Å represents the oxygen atoms of the surface hydroxy groups (O_S_H^+^). The integration of O_S_H^+^ density shows that 33.9% of surface O atoms are hydroxylated, and the value is slightly higher than that on the CeO_2_(111) surface. It should be noted that the CeO_2_(110) surface possesses twice the number of surface oxygen atoms as the CeO_2_(111) surface per primitive surface unit cell.

Two types of O*H_2_ adsorption structures are seen, and a small shoulder found at ∼1.6 Å represents the structure that involves a hydrogen bond with the surface O_S_ atom (see Fig. S7(a-ii)†). The other adsorption structure corresponding to the peak at ∼2.5 Å does not form a hydrogen bond with the surface O_S_ atom. The presence of two H_2_O* adsorption structures reflects the mobility of adsorbed water due to the low coordination number of the surface Ce atom. The density profile of O*H^−^ is higher than that of O*H_2_, and integrations of the O*H_2_ and O*H^−^ densities show that 38.0% and 67.2% of the Ce sites are coordinated by water molecules and hydroxide ions, respectively, and 63.9% of the water molecules are dissociated at the interface (it is noted that a single Ce site can be coordinated by multiple water molecules). This fraction is much higher than that on the CeO_2_(111) surface, which is consistent with the fact that the dissociative adsorption of a water molecule is much stronger than molecular adsorption on CeO_2_(110) compared to CeO_2_(111) (see Table S2†). The integration of the O densities of O*H_2_ and O*H^−^ yields a water coverage of 5.05 H_2_O nm^−2^, which is much lower than that in the previous work of DFT-MD simulations (7.52 H_2_O nm^−2^).^13^ One of the reasons could be the effects of dispersion correction, which is included in our simulation. The inclusion of dispersion correction results in a higher fraction of O*H^−^ ions at the interface, and these ions strongly adsorb on the surface Ce sites and electrostatically repel each other, leading to the reduced surface coverage of water molecules.

Three peaks are observed for H atoms, and the first peak at ∼1.0 Å corresponds to the surface hydroxy groups, and the second small peak at ∼1.5 Å indicates the H atoms of water molecules that are hydrogen bonded to the surface O_S_ atoms without coordinating to the surface Ce atoms (see Fig. S7(c-ii)†). The third prominent peak at ∼2.8 Å indicates the H atoms of O*H_2_, O*H^−^, or water molecules without coordination to the surface Ce atoms.

Some of the other decomposed density profiles, including OH^−^ and H_3_O^+^ ions that are not coordinated to Ce atoms, are given in ESI S.VII.†

### 3.3 Proton hopping and hydroxide ion transfer mechanisms

**Fig. 3:**
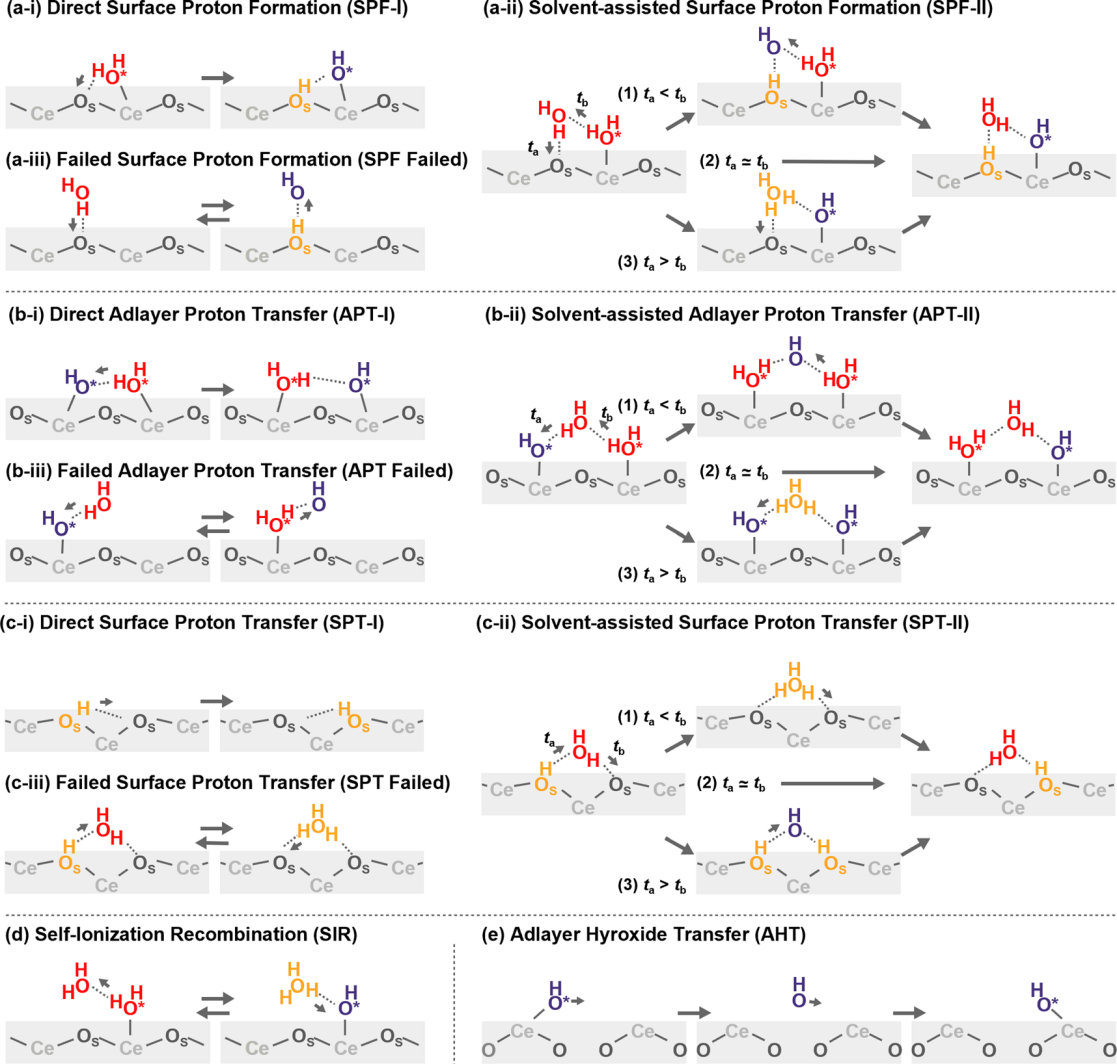
Schematic mechanisms of the proton/hydroxide ion transfers at surfaces. Species shown in orange have excess protons, and those shown in blue indicate hydroxide ions.

Proton hopping is quite active on the surface, and fast proton shuffling between the adsorbed water molecule and hydroxide ion was already reported in previous studies for the water/CeO_2_(111) interface.^12,13^ This unique property of the water/CeO_2_ interface could promote proton-assisted catalytic reactions in water. Based on the previous studies of other water/metal oxide interfaces, such as ZnO,^30,32^ we modify and extend the classification of mechanisms as follows (schematics are shown in Fig. 3).

In the following analysis, the proton hopping mechanisms that alter the surface protonation states and/or coordination states of the adlayer hydroxide ions are divided into four distinct classes. First, a proton hopping process between surface O atoms and an adsorbed water molecule O*H_2_ is referred to as surface proton formation (SPF; surface protonation, Fig. 3(a)), and its reverse process is called surface proton recombination (SPR, surface deprotonation). Next, a hopping process between an adsorbed water molecule O*H_2_ and a hydroxide ion O*H^−^ is called adlayer proton transfer (APT; Fig. 3(b)). Finally, a hopping process between a surface hydroxy group, O_S_H^+^, and a free O_S_ is termed surface proton transfer (SPT, Fig. 3(c)). Each class is further divided into two mechanisms: direct hopping (Type I, Fig. 3(a-i), (b-i), and (c-i)) and solvent-assisted hopping (Type II, Fig. 3(a-ii), (b-ii), and (c-ii)), wherein the latter mechanism an adjacent hydrogen-bonded water molecule participates in the hopping event. Furthermore, as shown in Fig. 3(a-ii), (b-ii), and (c-ii), each solvent-assisted hopping mechanism can be divided into three scenarios: proton relay with transient hydroxide ions (OH^−^ stepwise), concerted proton transfers (concerted), and proton relay with transient hydroniums ions (H_3_O^+^ stepwise). While the H_3_O^+^ stepwise in Fig. 3(b-ii-3) and OH^−^ stepwise in Fig. 3(c-ii-3) cases might be viewed as sequential self-ionization and recombination steps, we consider them as single processes in our analysis because they can be unified by introducing hopping timing, *t*_a_ and *t*_b_, as denoted in Fig. 3. We define transient ion lifetimes as Δ*t*_PT_ = *t*_b_ – *t*_a_, and thus positive and negative lifetimes indicate that of transient OH^−^ and H_3_O^+^ ions, respectively, in the case of SPF/SPR/APT-II, and *vice versa* in the case of SPT-II. Moreover, in the solvent-assisted proton transfer mechanism, a proton may return to the original surface oxygen or adlayer hydroxide ion before reaching another surface oxygen or adlayer hydroxide ion. These are considered as failures and are categorized as (Fig. 3(a-iii)) SPF Failed, (Fig. 3(b-iii)) APT Failed, (Fig. 3(c-iii)) SPT Failed, and (d) self-ionization recombination (SIR). Lastly, the rare occurrence of direct migration of an adlayer hydroxide ion to another Ce site is identified as adlayer hydroxide transfer (AHT, Fig. 3(e)), representing a case of the vehicle mechanism on the surface.

To proceed with the analysis of MD trajectories based on the above mechanisms, we identify links in the relayed transport of excess protonation states (O_S_H^+^ or H_3_O^+^) and proton holes (OH^−^) by tracking the exchange of protons between chemical species defined by the NNB assignment. For O atoms derived from water molecules, if only one hydrogen atom is assigned, this O atom is considered to possess a proton hole, and if three hydrogen atoms are assigned, this O atom is considered to be in an excess protonation state, H_3_O^+^. For O atoms of the CeO_2_ surface, if one hydrogen atom is assigned as a surface hydroxy group, this surface O atom is labeled as having an excess proton. By tracking the proton exchange and the change of the coordination states of the adlayer hydroxide ions, the hopping events occurring simultaneously on the surfaces and the relayed transport of the excess protonation states/proton holes can be identified. Here, when an O atom has multiple surface Ce atoms within the coordination threshold, the O atom is assigned to the nearest Ce atom. Note that even if a hydroxide ion desorbs/adsorbs from a Ce site before/after the hopping, such as depicted in ESI S.VIII,† we disregard the desorption event and categorize this occurrence as a proton hopping event to/from the adlayer hydroxide ion. Also, if a hydroxide ion migrates from interface regions, which are defined as heights of 6.5 Å and 6.0 Å from surface Ce atoms for CeO_2_(111) and (110), respectively, the relayed transport is omitted from our analysis. The heights have been determined by the peaks of oxygen density profiles that are hydrogen-bonded to the adlayer water molecules (see ESI S.VII†). The numbers of the hole trajectories that include such migrations are only 0.36 and 0.04 per ns per primitive surface unit cell for the water/CeO_2_(111) and (110) interfaces, respectively.

**Fig. 4:**
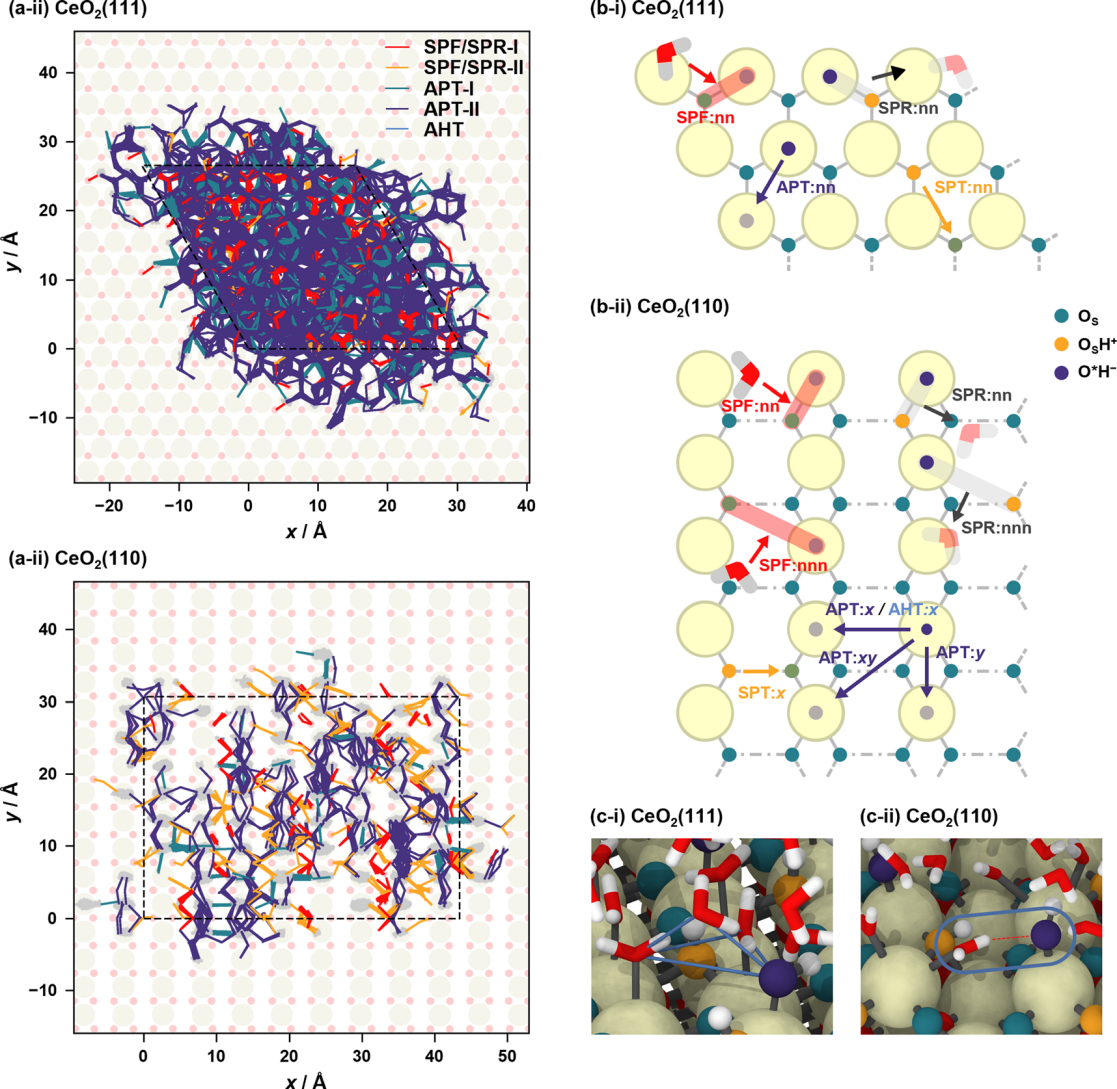
(a) Proton hole trajectories over the courses of 1 ns NNP-MD simulations. In the case of SPF/SPR, the lines connecting the corresponding O_S_ atom are also shown. The motions of hydroxide ions coordinated to surface Ce atoms are depicted as gray lines in the background. The dashed lines represent the simulation cells, and each trajectory is unwrapped relative to the simulation cell to illustrate the diffusion process of the proton hole. (b) Definitions of site pairs at which proton/hydroxide ion transfer events are classified. Here, “nn” and “nnn” mean the nearest neighbor and next nearest neighbor, respectively. (c) Snapshots showing characteristic adsorption structures on the (i) CeO_2_(111) and (ii) CeO_2_(110) surfaces. The blue lines in (c-i) are drawn to emphasize a typical tetrahedral structure near the surface.

Fig. 4(a) shows the proton hole trajectories during the 1 ns simulation that are connected by the proton hopping/hydroxide ion transfer events. In these figures, the trajectory lines corresponding to “failed” transfers are excluded. Movies of representative trajectories of the NNP-MD simulations are provided in the ESI.†Fig. 4(b) depicts the definitions of the surface site pairs at which proton hopping/hydroxide ion transfer events are classified and counted separately on CeO_2_(111) and (110). Table 1 presents the frequencies of each transfer event per nanosecond per primitive surface unit cell.

**Table 1:** Event frequencies calculated from 4 ns NNP-MD trajectories. The label “self” denotes proton transfers between oxygens coordinated to the same Ce site (see ESI S.VIII), and the label “other” represents proton/hydroxide ion transfers between pair sites that are not defined in Fig. 4. Here, event classes occurring with a frequency of less than 0.01 ns^−1^ per primitive surface unit cell (approximately twice during 4 ns simulations of *p*(8 × 8) surfaces) are omitted

CeO_2_(111)	ns^−1^ primitive surface^−1^			CeO_2_(110)	ns^−1^ primitive surface^−1^		
Reaction	Type	Pair	Frequency	Reaction	Type	Pair	Frequency
SPF	I	nn	25.93	SPF	I	nn	1.17
	II	nn	1.03			nnn	1.81
		Other	0.05		II	nn	0.35
	Failed		4.92			nnn	1.51

SPR	I	nn	25.99	Failed		18.06	
	II	nn	0.99	SPR	I	nn	1.14
		Other	0.03			nnn	1.80

APT	I	nn	60.90		II	nn	0.38
		Self	1.41	nnn		1.52	
	II	nn	128.27	APT	I	*x*	4.34
		Self	0.11			*y*	1.40
		Other	0.13			Self	0.44
	Failed		517.43		II	*x*	0.85

SIR			3.54	*y*	5.67		
AHT		nn	0.04			*xy*	0.38
					Self	0.03	
				Failed		60.21	
				SPT	I	*x*	2.48
					II	*x*	0.22
					Failed		0.38
				SIR			7.92
				AHT		*x*	0.03

On the CeO_2_(111) surface, proton hopping is quite active, occurring at about tens of picoseconds per primitive surface unit cell. APT-II occurs most frequently except for “failed” transfers and is accompanied by the migration of hydroxide ions between the Ce sites. On the CeO_2_(111) surface, molecular and dissociative adsorption on the CeO_2_(111) surface have similar adsorption energies as described above, and therefore, proton hopping occurs easily, which is reflected in the activity of APT. In addition, the water molecules adsorbed at the interface are found to have a characteristic tetrahedral configuration connected by hydrogen bonds to a solvent water molecule (see Fig. 4(c-i)). This structure promotes active proton hopping assisted by the solvent molecule. This quite active solvent-assisted proton transfer (APT-II) is one of the characteristics of the water/CeO_2_ interface; at a water/ZnO interface, solvent-assisted proton transfer events were reported only approximately 0.01 times per ns per primitive surface unit cell (41 times during 44 ns simulation with a double-sided symmetric *p*(6 × 8) slab model).^32^

On the CeO_2_(110) surface, the frequency of APT-II is much lower than that on the CeO_2_(111) surface. Since dissociative adsorption is more stable than molecular adsorption on CeO_2_(110), most of the surface Ce sites are covered by hydroxide ions. APT events require adlayer water molecules, and this high coverage of hydroxide ions reduces the transfer frequencies. There are basically two types of APT on the CeO_2_(110) surface, one along the *x*-axis and the other along the *y*-axis. The Ce–Ce distance is shorter along the *y*-axis, and APT-II along the *y*-axis is the most frequent on CeO_2_(110), where O*H_2_ and O*H^−^ species adsorbed on neighboring Ce atoms transfer protons through adjacent water molecules. In the case of APT-I, transfers along the *x*-axis are more common than those along the *y*-axis. This is because water molecules can adsorb on bridge-like sites along the *x*-axis, as shown in Fig. 4(c-ii), leading to a facile formation of hydrogen bonds with hydroxide ions. For SPF/SPR on CeO_2_(110), because the distance between the adjacent surface O_S_ atoms along the *x*-axis is short, the surface hydroxy group (O_S_H^+^) can be stabilized by forming a hydrogen bond with neighboring surface O_S_ atom, resulting in much lower activity than that on the CeO_2_(111) surface. We also found that the unique proton hopping mechanism occurs between the surface O_S_ atoms (SPT) on CeO_2_(110) and that AHT events occur only along the *x*-axis.

It is noted that the number of proton hopping events highly depends on the choice of the hydrogen assignment procedure. For comparison, the results of the analysis using the assignment based on the stable state picture (SSP)^79^ are given in ESI S.IX.† While the SSP can exclude rapid recrossing around the transition states of hopping from the count, we used the NNB assignment since we are interested in dynamics near transition states in the subsequent analysis.

**Fig. 5:**
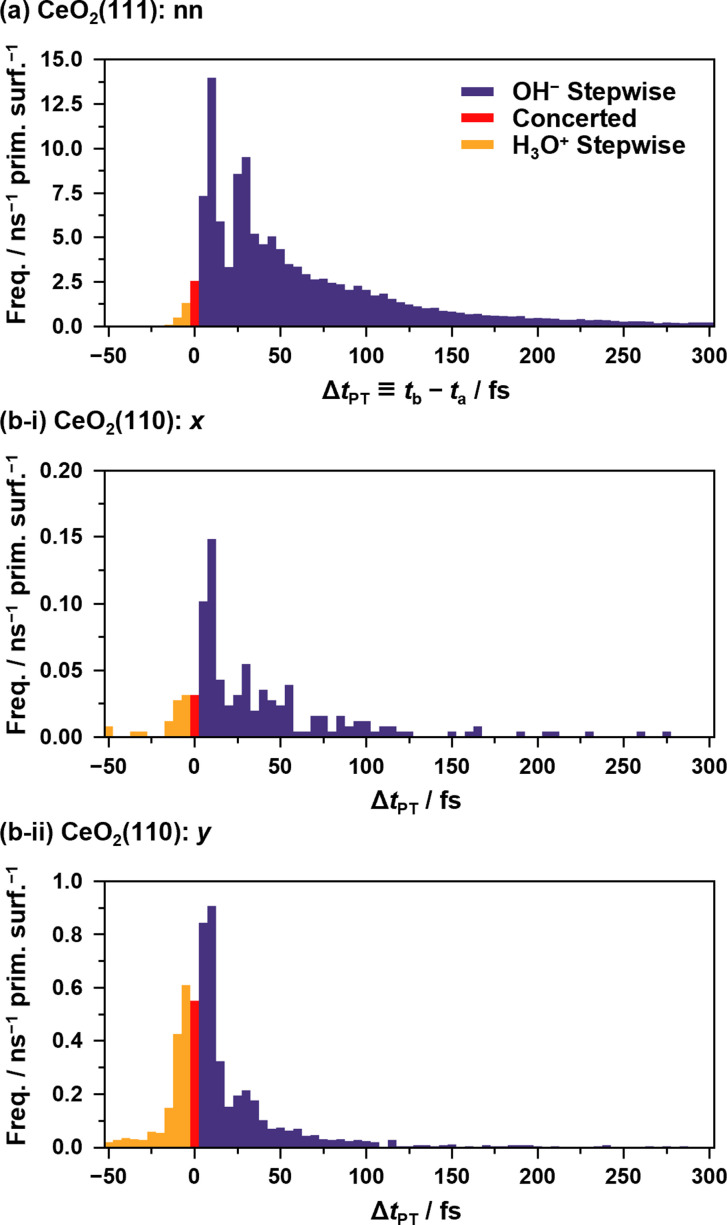
Histograms of Δ*t*_PT_ = *t*_b_ – *t*_a_ representing the transient lifetimes of OH^−^/H_3_O^+^ species during APT-II processes between (a) “nn” pairs on the CeO_2_(111) surface and between (b) “*x*”/”*y*” pairs on the CeO_2_(110) surfaces. *t*_a_ and *t*_b_ are defined in Fig. 3. By definition, negative values indicate the lifetimes of H_3_O^+^. In the case of APT-II with relays of multiple solvent water molecules, the summations of the lifetimes are used for counting. Note that APT-II with a single solvent water molecule is dominant: 98.5%, 93.5%, and 98.8% for the cases (a), (b-i), and (b-ii), respectively. The bin width of the histogram is 5 fs.

Fig. 5 shows the histograms of the lifetime of transient OH^−^ species in APT-II (see ESI S.X† for SPF/SPR/SPT-II). It is clear that there are at least two dominant components, peaks around 10 fs and 30 fs in each case, which is consistent with the concerted and stepwise mechanisms in a previous study.^12^ In the stepwise mechanism, the transient OH^−^ species derived from an assisting water molecule remains for a certain time before receiving a proton from another O*H_2_ species, whereas in the concerted process, a proton is transferred immediately after the formation of OH^−^. As discussed later in Section 3.5, however, these can be interpreted as coupling between the proton hopping and vibrational motion of water molecules rather than the presence of two distinct mechanisms.

### 3.4 Reaction rates and long-range proton/hydroxide ion transport determined using a semi-Markov model

In the previous subsection, the mechanisms and frequencies of proton hopping were analyzed in detail, but we did not provide the corresponding reaction rates and information on whether concatenated proton hopping events contribute to long-range transport along the surface *via* the Grotthuss-like diffusion mechanism. Multiple proton hopping events can occur between the same pair of a water molecule and a hydroxide ion, or a surface O_S_ atom, *i.e.*, “rattling” between the same donor and acceptor. In such cases, the probability of long-range transport is significantly reduced compared to what would be expected based on the frequencies of proton hopping events alone. To analyze the reaction rates of formation/transfer/recombination processes of protons/hydroxide ions, we develop a counting analysis to estimate the reaction rates from MD trajectories by assuming a stochastic process.

In this analysis, trajectories are regarded as realizations of a stochastic process where the state transitions are determined by a sequence of independent random jumps. First, we assume the elementary processes that occur in our system, *e.g.*, SPF/SPR on pair sites in Fig. 4(b). Then we count the pair states that have opportunities for the reactions, *e.g.*, adjacent site pairs that have an excess proton and a proton hole in the case of SPR, and we also count the number of reactions that actually occurred in the NNP-MD simulations. Because we have assumed that the transitions are independently determined, we can estimate the transition probabilities as the number of reactions per opportunity. The transition rates are calculated as the transition probabilities per unit time under the first-order approximation of the sampling interval, Δ*t*, and the confidence intervals of the rates within the stochastic process model can also be estimated by using statistics based on the binomial distribution. In this work, we use the Clopper–Pearson interval.^80,81^ In addition, by counting the situations of the surrounding site states around the pairs separately, we can incorporate the environmental dependence of the reaction rates (the so-called adsorbate–adsorbate lateral interaction effects in the case of on-lattice surface reaction models^82^). It is noteworthy that this counting approach can be regarded as an inverse analysis of the kinetic Monte Carlo (KMC) method,^82^ and by performing KMC simulations with the estimated reaction rates, we can reproduce stochastic trajectories. To keep the analysis within the first order of Δ*t* and to count only predefined reactions, if multiple reactions or undefined reactions occur in adjacent time frames, we ignore the reaction and omit the number of reaction opportunities that are calculated from the earlier time frame in the count. This prescription is valid as long as the occurrence of the events is stochastically independent and multiple reactions or undefined reactions do not cause the same result as one of the elementary reactions.

This counting analysis can be understood as a variant of the Markov state model (MSM) approach,^83^ extended to handle elementary reactions that occur simultaneously. However, there are several differences from the MSM approach. First, the MSM is usually applied to estimate the transition rates of the total state of the system, such as the conformational change of a protein and bimolecular reaction. Thus, the conventional MSM approach measures the duration of each state, while our approach counts the opportunities of each elementary reaction. Second, in the conventional MSM approach, the sampling time interval, the so-called lag time, is adjusted to coarse-grain the dynamics of the system to guarantee a Markovian process. In the case of our analysis, Δ*t* is upper bounded by the timescale of the elementary reactions, and we cannot ensure the validity of the stochastic process assumption by a time coarse-grained picture. As an alternative, we can check the reproducibility of the model (elementary reactions, environment dependence, *etc.*) by performing KMC simulations. Finally, in our analysis, the reaction rates can be time-dependent, and the corresponding stochastic process is no longer Markovian but rather semi-Markovian.^84^ This feature is useful for analyzing “rattling” dynamics in detail by considering the time interval dependence on the rates since the last event. However, if we consider the situation where the transition rate of an event depends on the time duration from the last events on the relevant sites, the entire process is not purely semi-Markovian because the normal semi-Markov process depends only on the time duration from the last event of the entire system. Hereafter, we refer to the class of this stochastic process as a “coupled” semi-Markov process.

In order to apply the counting analysis to the liquid/CeO_2_ interface, we perform a sort of coarse-graining to map the MD trajectories onto a series of lattice states of acid/base sites as follows. Here, the acid and base sites are the surface Ce atoms and O_S_ atoms, respectively. First, each trajectory of excess proton states and proton holes is assigned to the time series of transitions through proton hopping/hydroxide ion transfer events. Since “self” and “failed” transitions do not change the lattice states, these transitions are not counted. In addition, to count transitions, we need to decide the timing of the transition events because solvent-assisted proton hopping events have finite transition durations. Here, we employ the earlier timing of *t*_a_ and *t*_b_ shown in Fig. 3. Since this procedure assigns not only adlayer O*H^−^ but also solvent OH^−^ to surface acid sites, some sites may be doubly/triply occupied. These multiply occupied states are treated as such because they can be considered as a measure of the density of hydroxide ions around each site, and the number of assigned proton holes is referred to as the “number of hole occupations”.

Here, we discuss the results of the reaction rate analysis based on two different stochastic process models. In the first model, we do not distinguish between “rattling” events, where the corresponding excess protons/proton holes return to the previously occupied sites, and events where they move to another sites, and all reaction rates are time-independent constants. In the second model, we distinguish the “rattling” events from other events, and the reaction rates of the “rattling” events depend on the time interval from the corresponding last transition events. Hereafter, we refer to the first and second models as the coupled Markov state model (cMSM) and coupled semi-Markov state model (csMSM), respectively, because the first model can be regarded as a variant of the MSM on the acid/base lattice spaces, and the second model can be regarded as reflecting the non-Markovian nature of “rattling” transitions due to the kinetic motion of protons and the duration of hydrogen bond networks; for example, once a strong hydrogen bond is formed between an O atom pair, proton hopping between the pair could occur more frequently until the corresponding hydrogen bond is broken. In both models, we assume that the reaction rates of SPF/SPR/SPT depend on the number of protonated base sites around the corresponding O_S_ sites and that the reaction rates of SPF/SPR/APT/AHT depend on the “number of hole occupations” of the corresponding acid sites. For details on these models, see ESI XI.†

In order to check the validity of the above-mentioned two stochastic models, we compare the average hydroxylated surface O ratios, the survival functions and the mean square displacements (MSDs) of the proton holes, calculated from the NNP-MD trajectories and the KMC simulations based on the two models. Here, the proton holes instantaneously present during “failed” hopping events in the MD trajectories are excluded from the calculations. The conventional definition of the MSD for “immortal” particles is inappropriate for our cases since the number of “living” proton holes decays as a function of their lifetime. Here, we modify the definition to take the average only within the “living” proton holes, as2
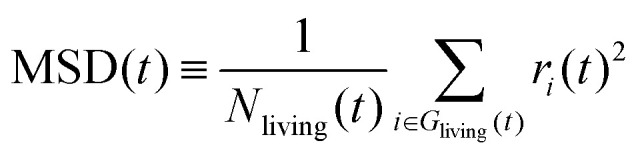
where *G*_living_(*t*) represents the set of proton holes “living” at lifetime *t,* and *N*_living_(*t*) is the number of them. Note that the accuracy of the MSD decays as a function of lifetime because the number of samples decays. We use the slopes at the average lifetimes to evaluate the diffusion constants.

**Fig. 6:**
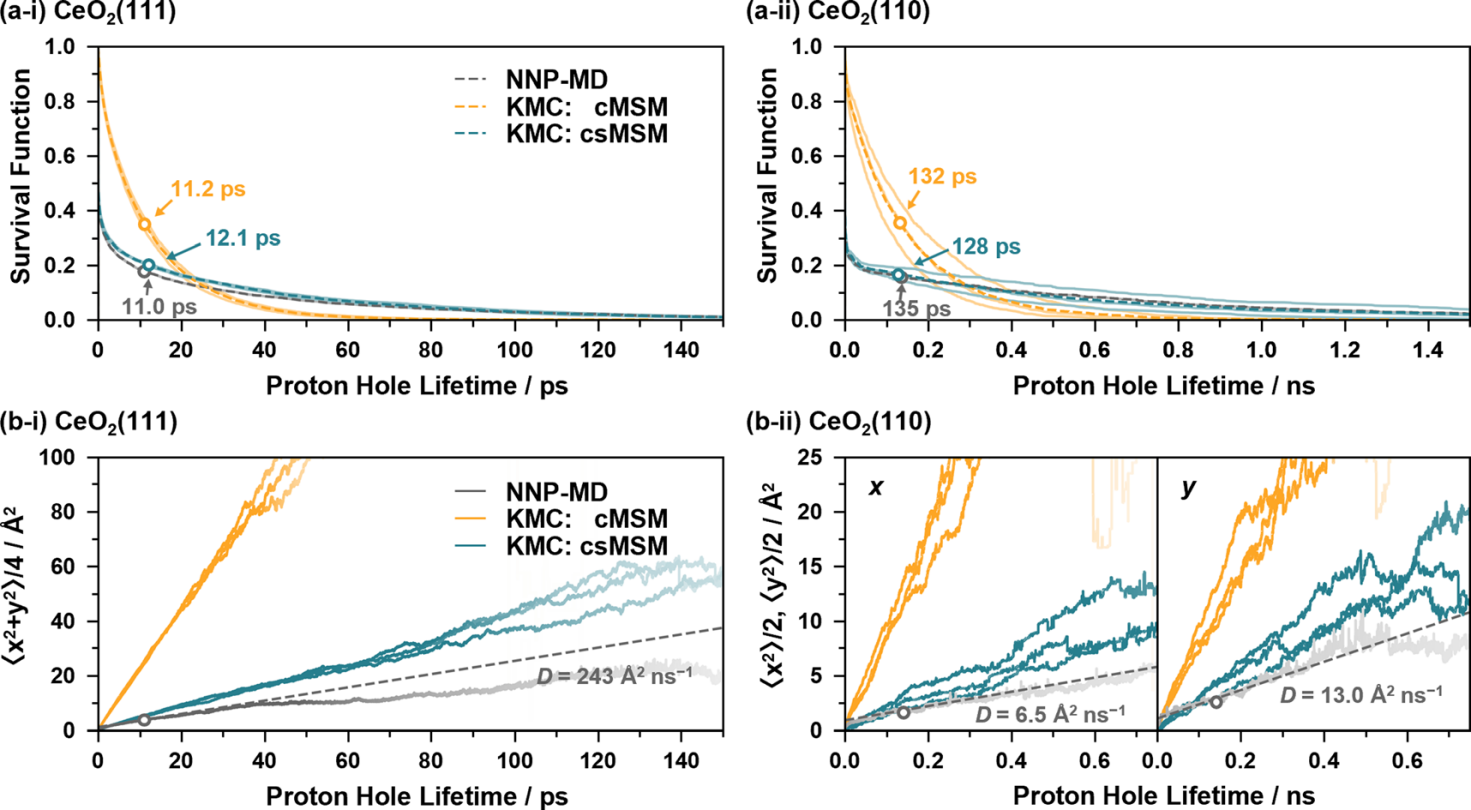
(a) Survival functions of proton holes relayed by proton hopping events, calculated from NNP-MD simulations and KMC simulations based on the reaction rates of cMSM and csMSM. The hollow circle on each dashed line represents the average lifetime. (b) Mean square displacement of the proton holes. Each curve is plotted thinner as the corresponding survival function becomes less than 5%. The dashed lines are linear extrapolations calculated from the slopes at the average lifetimes.

Fig. 6(a) and (b) show the survival functions and MSDs of proton holes relayed by proton hopping events, calculated from the 4 ns NNP-MD trajectories and from 4 ns KMC simulations based on the reaction rates of cMSM and csMSM. Here, the fixed timestep method^82^ is employed for the KMC simulations with a timestep of 5 fs. The lattice sizes are *p*(8 × 8), and the lattice site positions calculated from the averages of the NNP-MD simulations are employed to calculate the displacements of the proton holes. Three independent KMC simulations are performed for each model, varying the reaction rates within the 95% confidence intervals (see ESI S.XII†). The calculations of the survival functions include the lifetimes of the proton holes whose observations are truncated on the left and/or right by the simulation time. Note that, while the simulation time of 4 ns may not be sufficient to accurately evaluate the survival functions for CeO_2_(110) as proton holes have lifetimes in the nanosecond regime, it can be considered sufficient to compare NNP-MD and KMC results.

The KMC simulations, based on the reaction rates of cMSM and csMSM, yield hydroxylated surface O ratios of 29.0% and 29.1% for the CeO_2_(111) surface and 32.9% and 32.5% for the CeO_2_(110) surface, respectively, which show good agreement with the values obtained from the NNP-MD simulations of 29.8% and 33.8%. In addition, both models reproduce the average lifetime of the proton holes of the NNP-MD results as shown in Fig. 6(a), where the lifetime are estimated as 11.2 (132) and 12.1 (128) ps for cMSM and csMSM, respectively, while that of NNP-MD is 11.0 (135) ps for the CeO_2_(111) (CeO_2_(110)) surface. As seen, cMSM tends to underestimate the long tails of the proton hole lifetimes and to overestimate the diffusion constants of the proton holes. In contrast, csMSM reproduces the NNP-MD results of the survival functions well and show better agreement with the diffusion behavior of the NNP-MD results. Due to the “rattling” of the SPF/SPR processes, the survival functions exhibit a rapid decay around time zero. While the csMSM models still tend to overestimate the diffusion constants, the deviation can be considered close to the estimation range of the csMSM analysis as shown in Fig. 6(b). The lower diffusion constant of csMSM compared to that of cMSM reflects the importance of considering “rattling” transitions due to the finite lifetimes of hydrogen bond networks, which increase the possibilities of proton hopping reversions and provide “memory effects” to the interface reactions. Also, a comparison of the diffusion constants of CeO_2_(111) and (110) clearly demonstrates the long-range proton hole (hydroxide ion) transport ability of CeO_2_(111).

**Fig. 7:**
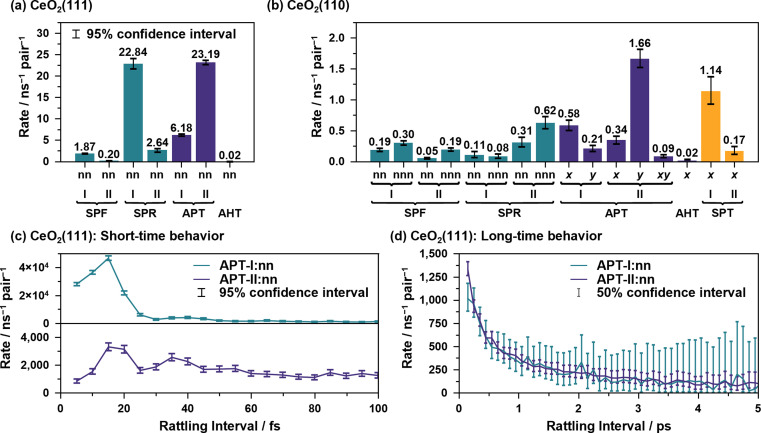
Reaction rates of non “rattling” components calculated by the csMSM analysis for the (a) CeO_2_(111) and (b) CeO_2_(110) surfaces. (c) Short time behavior of the “rattling” APT reaction rates for the CeO_2_(111) surface. (d) Long time behavior of the “rattling” APT reaction rates in (c), where the values are averaged every 100 fs.

Fig. 7(a) and (b) illustrate the average reaction rates of the non “rattling” reactions, *i.e.*, the reaction rates for the reaction on new site pairs, estimated by using csMSM (see ESI S.XIII† for the values depending on the surrounding state). These can be considered as the fundamental quantities describing the long-time and diffusive behavior of the proton hole transfer. As shown in Fig. 7(a), APT-II is still more active than APT-I even when the “rattling” contributions are eliminated, and it is the dominant mechanism for long-range proton hole transfer on CeO_2_(111). While the summation of the APT reaction rates on the *y*-axis is larger than that on the *x*-axis on the CeO_2_(110) surface, as shown in Fig. 7(b), the difference of the diffusion behavior in the directions is small, as shown in Fig. 6(b-ii). This would be explained by the difference in lattice sizes in the direction along the *x*/*y* axes and by the difference in “rattling” reaction rates.

The reaction rates obtained by the csMSM approach can be regarded as a result of the two-step coarse-graining strategy from the DFT calculations. The first step is the coarse-graining of the electronic structures by using the NNP construction through the enhanced sampling of atomic configurations, and the second step is the coarse-graining of the atomic configurations into lattice states using the NNP-MD simulation and counting analysis based on csMSM. Note that while we did not explicitly count the molecular adsorption/desorption processes of water, the reaction rates include the effective timescale of these processes. In other words, the molecular adsorption states were coarse-grained in our models. While this may be one of the reasons why the proton hole diffusion is overestimated in our csMSM models, effective reaction constants are helpful to understand the proton–proton hole pair formation/diffusion/recombination processes in a simple way.

To close this subsection, we discuss the short- and long-time behavior of the time-dependent reaction rates of the “rattling” APT reactions. Fig. 7(c) and (d) show the average results of the short-time and long-time behavior for the water/CeO_2_(111) interface. The values for other elementary reactions and those for the water/CeO_2_(110) interface are given in ESI S.XIV.† Clearly, oscillatory behavior, with time periods of approximately 24 fs and 18 fs for APT-I and APT-II reactions, respectively, and picosecond scale decaying behavior are shown. These can be interpreted as couplings to the vibrations of the adlayer/solvent water molecules and the timescales of the destruction of the local hydrogen bond network. In the next subsection, we discuss such vibrational motions and hydrogen bond lifetimes, which were coarse-grained in our stochastic model analysis, for comparison.

### 3.5 Dynamics coupled to proton hopping

The hydrogen bond lifetime is examined to investigate hydrogen bond dynamics at the interface. We consider the existence of a hydrogen bond if the distance between O_D_ and O_A_ (O_D_ is a donor and O_A_ is an acceptor of a proton H) is less than 3.5 Å and the angle ∠O_A_O_D_H is less than 30°, following previous work.^85^ To evaluate the lifetime of the hydrogen bonds, we calculate the following correlation function,3
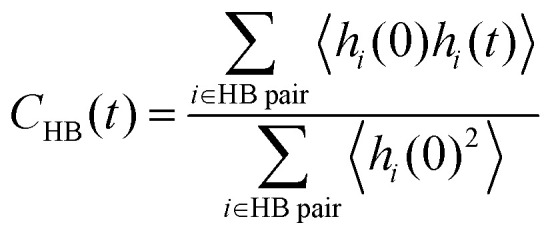
where *h*(*t*) = 1 if a hydrogen bond exists continuously up to time *t*; otherwise, *h*(*t*) = 0, and the sum runs over hydrogen bond pairs that exist at *t* = 0.^86,87^ This is the so-called “continuous” hydrogen bond lifetime correlation function. In order to distinguish between contributions due to changes in the hydrogen bond network and those due to proton hopping, we introduce two different types of hydrogen bond pair counting. In the first type, the atomic combination of the hydrogen and hydrogen bond donor/acceptor is strictly distinguished in the count. In the second type, only hydrogen-bonded oxygen pairs are counted without distinguishing the donor/acceptor relationship. We refer to these correlation functions based on the first and second types of counting as *C*^Strict^_HB_(*t*) and *C*^O pair^_HB_(*t*), respectively. In the former, if a proton hopping event occurs and the donor and acceptor relationship is swapped, the hydrogen bond is considered broken, while in the latter, the bond is maintained. Note that because we have adopted the NNB assignment, the hydrogen is always considered to be bonded to one of the O atoms, and the “hydrogen-bonded O pair” count is not interrupted during a hopping event. In the case of bulk water, both correlation functions give the same result.

To examine the hydrogen bond network of water molecules near the interface, we count only hydrogen bonds in which donor and/or acceptor oxygen atoms are coordinated to surface Ce atoms at *t* = 0. For comparison, we conducted a 1 ns bulk water NNP-MD simulation with 800 water molecules after equilibration at a temperature of 360 K and a density of 1.00 g cm^−3^ by using one of our NNPs. The average lifetime of the bonds is calculated as the time integration of *C*_HB_(*t*). Note that the value of the lifetime is strongly dependent on the definitions of the hydrogen bond and lifetime correlation function, the lifetime estimation method, and the simulation conditions, *e.g.*, temperature.

**Fig. 8:**
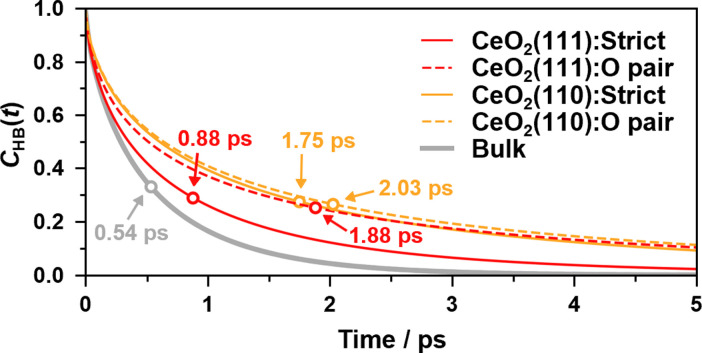
The continuous hydrogen bond lifetime correlation functions, *C*_HB_(*t*), of adlayer water molecules and bulk water molecules. The sampling resolution is 5 fs. Lifetimes are shown as hollow circles.

Fig. 8 shows the hydrogen bond lifetime correlation functions at the water/CeO_2_(111) and water/CeO_2_(110) interfaces and in bulk water. While the lifetimes of the adlayer water molecules are longer than that of the bulk water due to their restraint on the surfaces, the “strict” correlation function of the water/CeO_2_(111) interface system decays faster than that of the water/CeO_2_(110) system. This reflects the fact that proton hopping is more active at the water/CeO_2_(111) interface, and thus, the local hydrogen bond networks are destroyed by the hopping. This is confirmed by comparing the “O pair” correlation functions, where the water/CeO_2_(111) and water/CeO_2_(110) interfaces exhibit similar decay profiles. We note that the decay profile of the “rattling” reaction rates has a similar decay timescale (see Fig. 7(d)) to that of the hydrogen bond correlation at the interfaces. This means that the “rattling” behavior, as expected, is closely related to the local hydrogen bond networks.

**Fig. 9:**
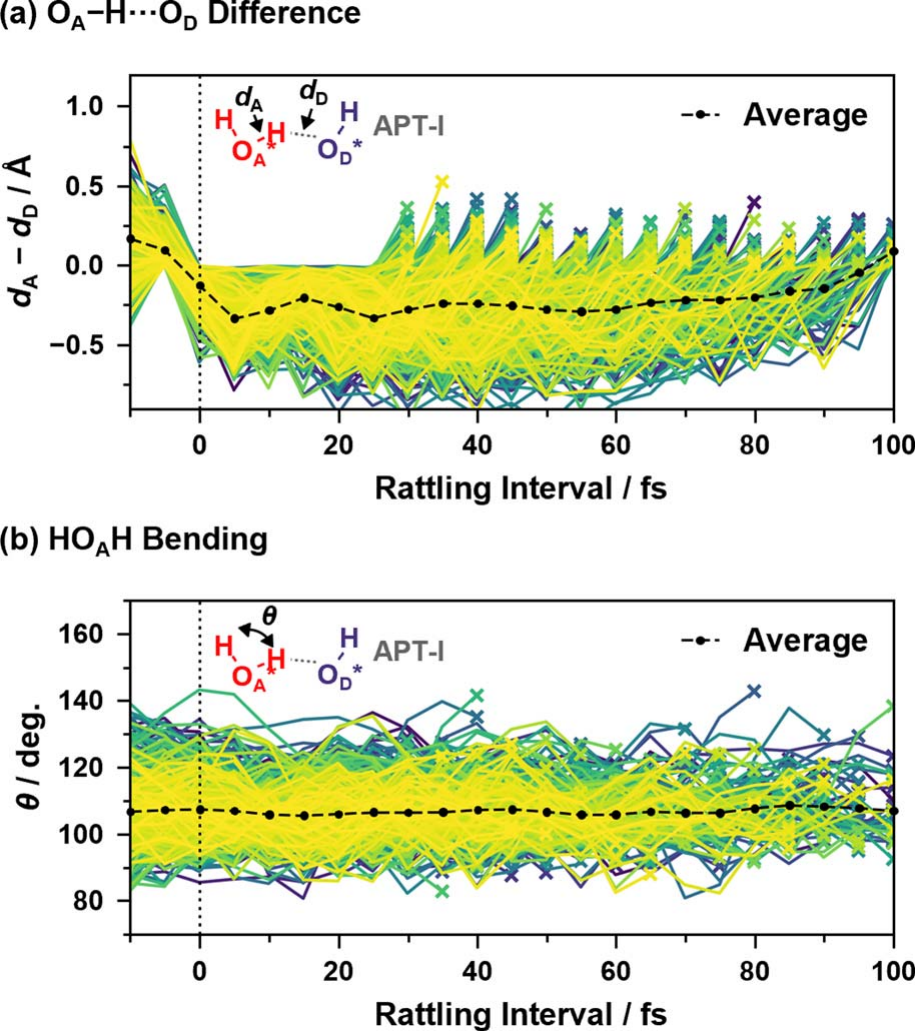
(a) Difference between the length of O_A_*–H and that of H–O_D_* and (b) HO_A_*H bending angle as a function of time since the last hopping events during “rattling” APT-I processes. Lines of different colors represent different samples, and the cross mark on each line indicates that the “rattling” proton hopping is occurring at that time. Here, the events with a “rattling” interval of 25–100 fs are included, and the averaging calculations are performed as in eqn (2).

Fig. 9 shows two coordinates during the “rattling” APT-I processes when an oxygen atom O_A_* receives a proton and pushes it back to the oxygen atom that originally had the proton, O_D_*. Here, in order to verify that the origin of the peaks in the reaction rates shown in Fig. 7(c) is a vibrational behavior rather than two or more distinct mechanisms, we include in the sample only processes that have a “rattling” interval of 25–100 fs; if the peak around 15 fs in the top panel of Fig. 7(c) had a different mechanism than other peaks, results calculated from 25–100 fs data would not have a vibrational peak around 15 fs.

As illustrated in Fig. 9(a), the difference between the length of O_A_*–H and that of H–O_D_* shows a clear correlation with the “rattling” proton hopping and has a peak around 15 fs. This indicates the transient presence of vibrational motions coupled to the proton hopping in the APT-I processes, with an average vibrational time period of ∼24 fs, much slower than the normal OH stretching of ∼10 fs (see ESI XVI†). While we also examined the coupling between water bending motion and hopping processes in Fig. 9(b), as the period of bending is close to the period that appears in reaction rates, no clear correlation is seen.

Although it is not a straightforward task to find the transient modes corresponding to the APT-II processes because they consist of relays between three or more water molecules, the result of the csMSM analysis indicates that such modes exist in the processes. It is noteworthy that we have already encountered similar time periods in the transient OH^−^ lifetime histograms of the APT-II processes, as shown in Fig. 5. This can also be explained as follows. When the timing of the modes coupled to the surface proton hopping and the actual hopping coincide, they cause nearly concerted processes. If the hopping reaction is delayed by one or more periods, the reactions become stepwise processes. Thus, the time-dependence analysis based on the csMSM approach clearly shows the detailed dynamics of proton hopping quantitatively. Note that csMSM is helpful not only for discussing time-dependent reaction rates but also for extracting the constant (Markovian) part apart from the time-dependent part. Also, while the number of proton hopping events is very sensitive to the proton-assignment procedure, the Markovian part of reaction rates calculated from the csMSM approach is less sensitive to it (see ESI S.XVI†). Therefore, the reaction rate constants we obtained can be considered intrinsic regardless of the details of the counting method, such as the threshold for bonding.

## 4. Conclusions

In this work, DFT-based NNP-MD simulations were performed to gain insight into the structural properties, dynamic behavior, and proton and hydroxide ion transport phenomena of the water/CeO_2_ interface. Unprecedented reactive MD simulations at the interface were performed by employing NNPs with a nanosecond timescale, extending the size and timescale of DFT-MD simulations. The construction of reliable NNPs is the key to running stable NNP-MD simulations, and the DFT-MD simulations with properly designed enhanced sampling methods and refinement schemes were effective in accumulating training data. In particular, the inclusion of configurations with complex proton hopping events is crucial for the construction of reliable NNPs to obtain statistically converged results with respect to proton hopping events.

Large-scale and long-time NNP-MD simulations provided more detailed information on the structure and the proton hopping mechanisms at the interface. The intricate dynamics of water dissociation and proton hopping events were analyzed in detail, and then the corresponding reaction rates were extracted under the assumption of a stochastic process, csMSM. To estimate the reaction rates with sufficient confidence, the NNP-MD simulations with large system sizes and nanosecond timescales were essential. We found that the CeO_2_(111) surface is a quite active facet for the transport of protons and hydroxide ions, leading to shorter lifetimes and larger diffusion constants of the proton holes before recombination with the hydroxylated surface oxygen atoms. The coupling between hopping events, vibrational motions, and hydrogen bonding lifetimes in the water/CeO_2_ systems was quantitatively demonstrated by the time-dependent analysis of the reaction rates.

The role of oxygen vacancies at the water/CeO_2_ interface and the interface with other solvents, such as alcohols, are also important topics to be investigated in the future. It is also interesting to investigate the effect of pH since the proton or hydroxide ion transfer will be significantly affected by pH. The nuclear quantum effects of hydrogen can be significant for proton hopping events, especially in “rattling” reactions, which is currently outside the scope of this work. Structural properties that account for nuclear quantum effects can be obtained by exploiting the imaginary time path integral formulations, although some approximate treatment would be required to gain insight into the dynamical properties. Extensions of the cMSM/csMSM analysis, *e.g.*, including nuclear quantum effects, dealing with solvents other than water, the molecular adsorption states, second hydration layers, and reactions in bulk region, employing data mining approaches for environment dependence analysis, are also interesting topics. Finally, more accurate DFT or wavefunction based simulations should be conducted in the future to obtain more reliable results.

Liquid/metal oxide interfaces are relevant in a variety of research disciplines, and NNP-MD simulations capable of running large-scale and long-time simulations will become a powerful tool to explore the microscopic structures, dynamics, and chemical reactions at the liquid/solid interface. The information obtained this way will be invaluable for the design of new materials in a wide range of technological applications.

## Data availability

The data that support the findings of this study are available from the corresponding author upon reasonable request.

## Author contributions

T. K. and T. I. performed calculations. T. I. and A. N. conceived and supervised this work. T. K., T. I., and A. N. analyzed the data. The manuscript was drafted with the support and contribution from all authors.

## Conflicts of interest

The authors declare no competing financial interest.

## Supplementary Material

SC-015-D4SC01422G-s001

SC-015-D4SC01422G-s002

SC-015-D4SC01422G-s003
