# Supplementary material for: Long-range proton and hydroxide ion transfer dynamics at the water/CeO2 interface in the nanosecond regime: reactive molecular dynamics simulations and kinetic analysis
Source: Chem Sci. 2024 Apr 2;15(18):6816–32. doi: 10.1039/d4sc01422g (PMC11077578; doi:10.1039/d4sc01422g)
Supplement: SC-015-D4SC01422G-s003 [file SC-015-D4SC01422G-s003.pdf]

Electronic Supplementary Material (ESI) for Chemical Science.
This journal is © The Royal Society of Chemistry 2024

Supporting Information

Long-range proton and hydroxide ion transfer dynamics at the water/CeO_2 interface in the nanosecond regime: reactive molecular dynamics simulations and kinetic analysis

Taro Kobayashi,[1] Tatsushi Ikeda,[1,*] and Akira Nakayama[1,*]

[1]*Department of Chemical System Engineering, The University of Tokyo, Tokyo 113-8656, Japan*
Corresponding Author*: t_ikeda@chemsys.t.u-tokyo.ac.jp, nakayama@chemsys.t.u-tokyo.ac.jp

S.I. Biased MD and Temperature Accelerated MD

S.I.1 Biased MD

The Biased MD (BMD) is introduced by adding a bias potential, which is a function of collective variables (CVs), to the potential energy of the system. These CVs are chosen so that the corresponding energy barrier can be easily overcome within the timescales of DFT-MD simulations. In this study, BMD was employed to promote proton hopping between water molecules and surface O atoms. For this purpose, the minimum distance between H atoms and surface O atoms was taken as CV, where it was defined as

$$S = \frac{\beta}{\ln \sum_{i}^{N} \exp\left(\beta / s_i\right)} . \tag{1}$$

Here, s_i is the distance between the reference atom and atom i in the system. In this study, an approximate free energy curve that was a function of the minimum distance between a particular O atom of the $CeO_2(111)$ surface and H atoms of water molecules was first obtained by the umbrella sampling prior to the BMD simulation, and this energy curve with the opposite sign was used to design a proper bias potential. The parameter β was set to 500 Å. This bias potential flattens the barrier of proton hopping between water molecules and surface O atoms.

Since the approximate free energy curve does not need to be the precise true free energy curve of the system as long as the curve flatten the barrier, we roughly estimated the function by using a smaller simulation cell without dispersion correction. The free energy curve was obtained by the umbrella sampling method employing a $CeO_2(111)$ slab model that had a surface area of $p(3\times3)$ and three O−Ce−O tri-layers, and 50 water molecules were placed over the surface. The DFT-MD simulation of 10 ps was performed for each umbrella window, and a total of 10 windows were used, where a quadratic potential centered at predefined points was applied with a force constant k ranging from 2 to 60 eV/Å^2 depending on windows (see Table S1). During the umbrella sampling, the bottom O−Ce−O tri-layers were fixed, the mass of hydrogen was replaced with that of deuterium, and the timestep and temperature were set to 1.0 fs and 360 K, respectively. The biased probability distributions from different umbrella windows were reweighted and patched using the weighted histogram analysis method (WHAM).[1] The computed free energy curve is shown in Figure S1, along with the histograms of the minimum distance for each window.

Table S1. Center of restraints $s^{(k)}$ and force constant k.

$s^{(k)}$ / Å	k / eV Å^{-2}
1.00	2.073
1.10	62.19
1.15	41.46
1.20	41.46
1.25	41.46
1.30	41.46
1.40	20.72
1.50	20.72
1.60	5.182
1.70	1.036

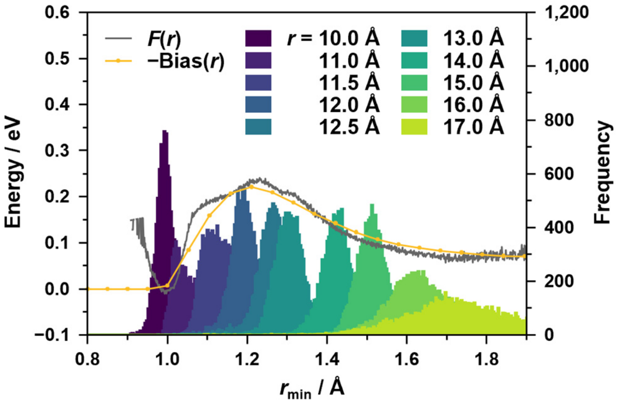

Figure S1. Roughly estimated free energy curve, $F(r)$, as a function of the minimum distance between one of the surface O_S atoms and all H atoms of water molecules. The actual bias function with a negative sign and the histograms obtained by the umbrella sampling are also shown with a bin width of 10^{-3} Å.

After estimating the free energy curve, a bias function that roughly cancels the energy barrier was designed to smooth out unnecessary detailed profiles of the curve (see Figure S1). This potential was applied to all surface O atoms in the BMD simulations. The same bias potential was used for the BMD simulations at the water/CeO_2(110) interface. The PLUMED package[2] interfaced with CP2K was used for the enhanced sampling.

S.I.2 Temperature Accelerated MD

In the temperature accelerated MD (TAMD), which is also called driven adiabatic free energy dynamics (d-AFED),[3][4] the following Lagrangian is introduced,

$$L_{\mathrm{TAMD}}(\mathbf{R},\dot{\mathbf{R}},\mathbf{s},\dot{\mathbf{s}}) = L_0(\mathbf{R},\dot{\mathbf{R}}) + \sum_{\alpha=1}^{n}\left[\frac{\dot{s}_\alpha^2}{2\mu_\alpha} - \frac{k_\alpha}{2}\left(S_\alpha(\mathbf{R}) - s_\alpha\right)^2\right] \tag{2}$$

Here, L_0 is the unbiased Lagrangian of the system, and a set of auxiliary variables $\{s_\alpha\}$ is introduced that is coupled to the CVs $\{S_\alpha\}$ by a harmonic potential with force constant k_α. The auxiliary subsystem $\{s_\alpha\}$ is thermostatted to high-temperature T_h, while the real system is thermostatted to a much lower system temperature T_0, which was set at 360 K in this study. The mass of auxiliary variables $\{\mu_\alpha\}$ is set to much larger than the atomic mass of the system so that the adiabatic decoupling of the auxiliary variables from the degrees of freedom in the system is guaranteed.

In this work, we introduced the coordination number as CVs. The ordinary coordination number between two atom types is defined as

$$\mathrm{CN}[X-Y] = \frac{1}{N_X}\sum_{i\in X}\sum_{j\in Y} c_{ij} \tag{3}$$

where N_X is a number of atoms with atom type X, and c_{ij} is a so-called switching function and represented as

$$c_{ij} = \frac{1-\left(\dfrac{d_{ij}}{R_0^{XY}}\right)^p}{1-\left(\dfrac{d_{ij}}{R_0^{XY}}\right)^{p+q}} \tag{4}$$

where d_{ij} is the distance between atom i and j belonging to the atom types X and Y, respectively, and p and q are parameters to define the shape of the switching function. R_0^{XY} is a cutoff bond distance between atom types X and Y. Based on this coordination number, an associated coordination number is defined as

$$\mathrm{CN}[X-Y-Z] = \frac{1}{N_X}\sum_{i\in X}\sum_{j\in Y} c_{ij}\sum_{k\in Z} c_{jk} \tag{5}$$

where the indices i, j, and k run over all atoms of atom types X, Y, and Z, respectively. We used the associated coordination number defined as CN[Ce–O–H] to promote proton hopping of water molecules or hydroxide ions coordinated to surface Ce atoms. Here we introduced independent CVs for each of the surface Ce atoms, resulting

in a total of 16 CVs for TAMD simulations for each surface. The parameters of $p = q = 6$ were employed, and cutoff bond distances between Ce−O and O−H were set to 2.8 Å and 1.2 Å, respectively. The TAMD simulations were performed with $\mu_\alpha = 50.0$ amu, $k_\alpha = 2.0$ a.u., and the temperature for the auxiliary variables was set to T_h = 2,000 K.

S.II. Construction and Refinement of NNP

The size of the embedding network (DNN for symmetric function) was set to (10, 20, 40, 80). The fitting network had hidden layers of size (240, 240, 240). The activation function was tanh. The number of training batches was set to 10^6 to ensure sufficient convergence of the loss function, and the batch size was set to 1. The learning rate started from 5×10^{-3} and finally decayed to 1.1×10^{-8}.

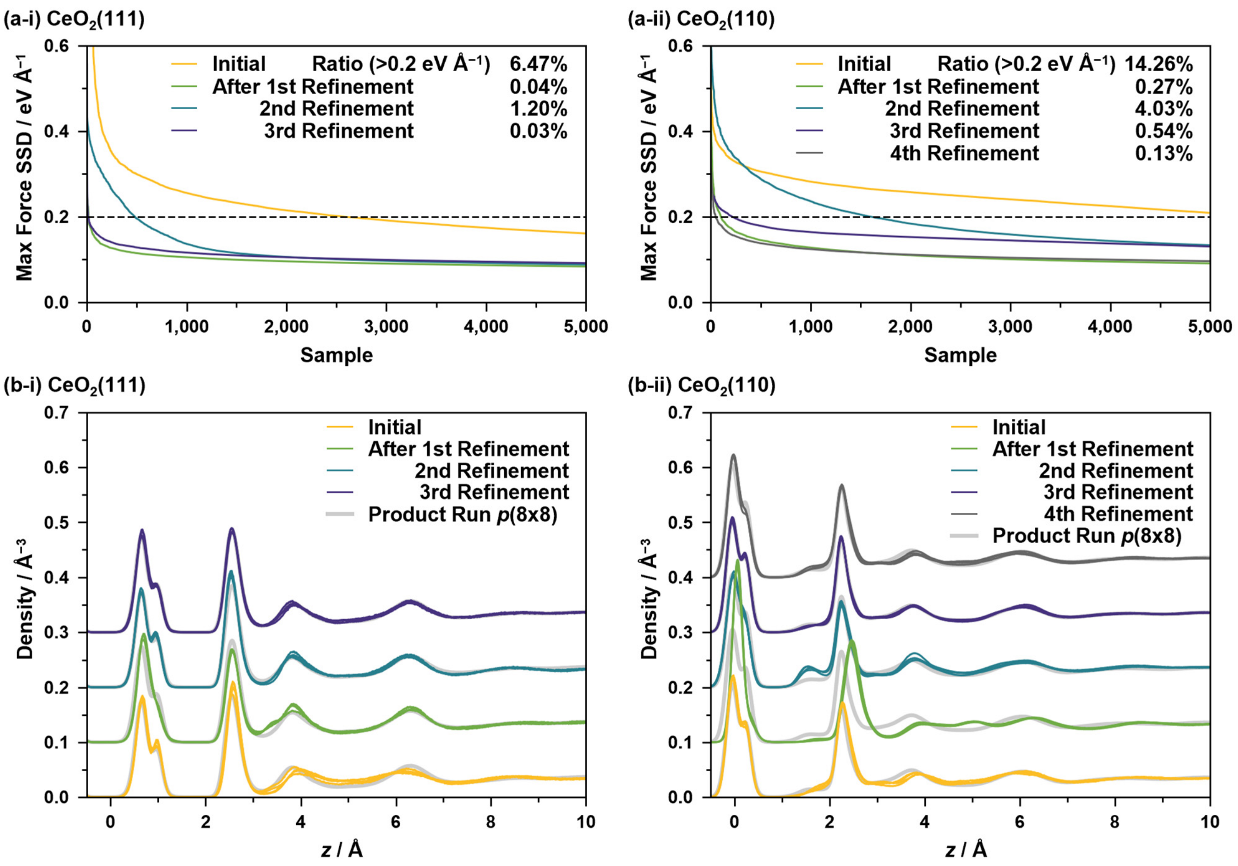

Figure S2. (a) Top 5,000 values of the maximum of the SSD of the forces, calculated from the structures of the MD simulation during each refinement step. Ratios of structures exceeding 0.2 eV/Å are also shown. (b) Convergence of the oxygen density distributions. Each density distribution for the initial/refinement step has four curves, which correspond to the four independent trajectories by using different NNPs. The curves are shifted for better visibility, and the density distribution calculated from the product run is also plotted for each shift level. In order to check the convergence in the third refinement for the CeO_2(110) system, the results calculated from the fourth refinement step are also shown, while the NNPs from this step are not used in the product run.

In Figure S2, the improvements in NNP accuracy and oxygen density distribution deviation during the

refinement procedure are shown. Here, the maximum sample standard deviation (SSD) of the forces is defined as

$$\max_{i,\alpha}\left\{\sqrt{\frac{1}{4}\sum_{s=1}^{4}\left(F_{i,\alpha}^{\mathrm{NNP}i}-\overline{F_{i,\alpha}^{\mathrm{NNP}}}\right)^{2}}\right\}. \tag{6}$$

S.III. Adsorption Structure of a Water Molecule on the CeO_2 Surface

The adsorption structures of a single water molecule were calculated by geometry optimization for two low-index surfaces. In calculating the energies of gas-phase molecules, the cubic simulation cell of $a = b = c = 15.0$ Å was used. For the calculation of the energies of slab models, the simulation cell was modified to have $c = 30$ Å from that described in the main text to ensure a vacuum space of at least 15 Å. For geometry optimization, the forces on all atoms were minimized to less than 0.02 eV/Å (4.5×10^{-4} hartree/bohr). The adsorption energy of a water molecule E_A to the surface was calculated according to

$$E_{\mathrm{A}} = E_{\mathrm{water+surf}} - E_{\mathrm{water}} - E_{\mathrm{surf}} \quad (7)$$

where $E_{\mathrm{water+surf}}$ is the total electronic energy of the water-surface system, while E_{surf} and E_{water} are the energies of the pristine CeO_2 slab model and an isolated water molecule, respectively. In this definition, the more negative value of adsorption energy indicates a stronger binding to the surface. The adsorption energies are given in units of eV in Table S2, and the adsorption structures are shown in Figure S3.

Table S2. Adsorption energies of a water molecule in molecular and dissociative states on the CeO_2(111) and (110) surfaces.

	Molecular	Dissociative	Calc.	Ref.
CeO_2(111)	−0.74	−0.75	PBE+U with dispersion	this work
	−0.63	−0.66	PBE+U	[5]
	−0.67	−0.61	PBE+U	[6]
CeO_2(110)	−1.06	−1.24	PBE+U with dispersion	this work
	−1.44	−1.76	PBE0	[7]
	−0.79	−1.03	PBE+U	[5]
	−0.85	−1.12	PBE+U	[8]

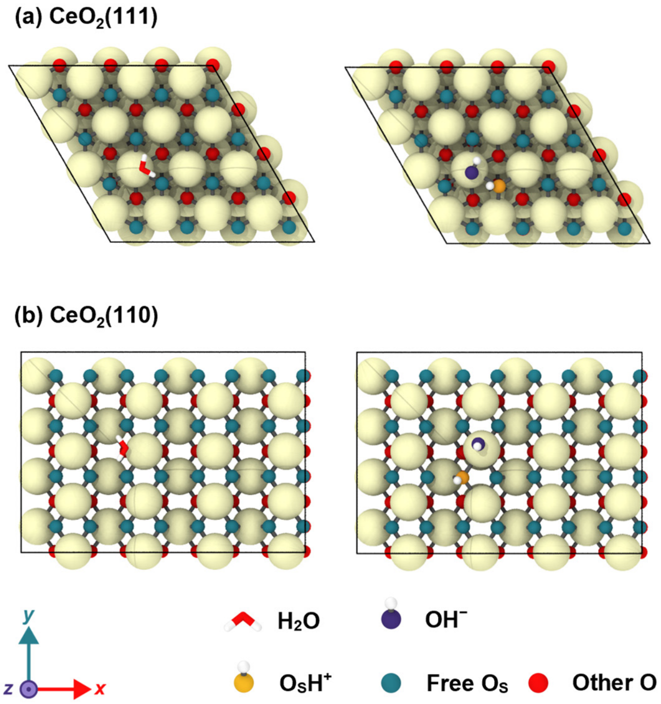

Figure S3. Adsorption structures in molecular and dissociative states on the (a) $CeO_2(111)$ and (b) $CeO_2(110)$ surfaces.

S.IV. Degree of Surface Hydroxylation as a Function of Time

Figure S4 shows the ratio of various surface species as a function of time, starting from undissociated water molecules by NNP-MD simulations for *p*(8×8) surfaces. The definition of the surface hydroxy groups, O_SH^+, surface hydroxide ions, O^*H^-, and adsorbed water molecules, O^*H_2, are given in the main text. It should be noted that, as our NNPs have been specifically trained to evaluate thermal equilibrium states, the reliability of the relaxation processes far from equilibrium is not guaranteed.

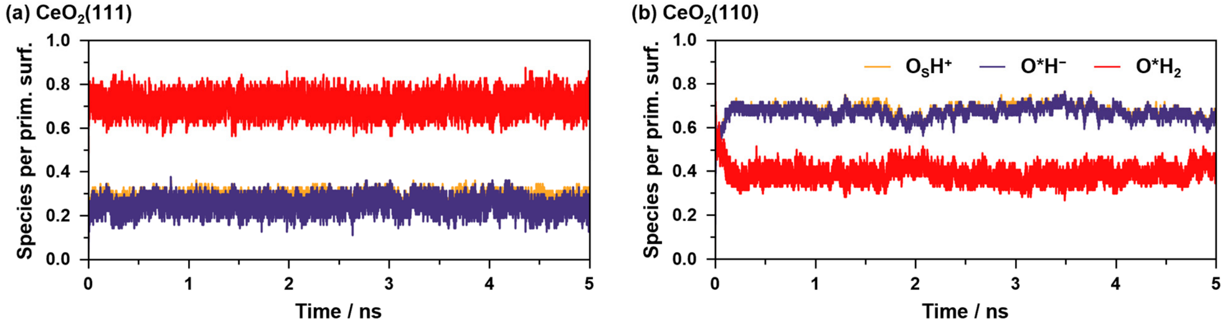

Figure S4. Ratio of surface/adlayer species per primitive surface unit cell as a function of time.

S.V. Snapshots of NNP-MD Product Run

Figure S5 shows snapshots of the trajectories of NNP-MD product run. A water molecule escaped from the water layer into the vacuum region in the snapshot of the water/CeO_2(110) interface was removed to avoid confusion.

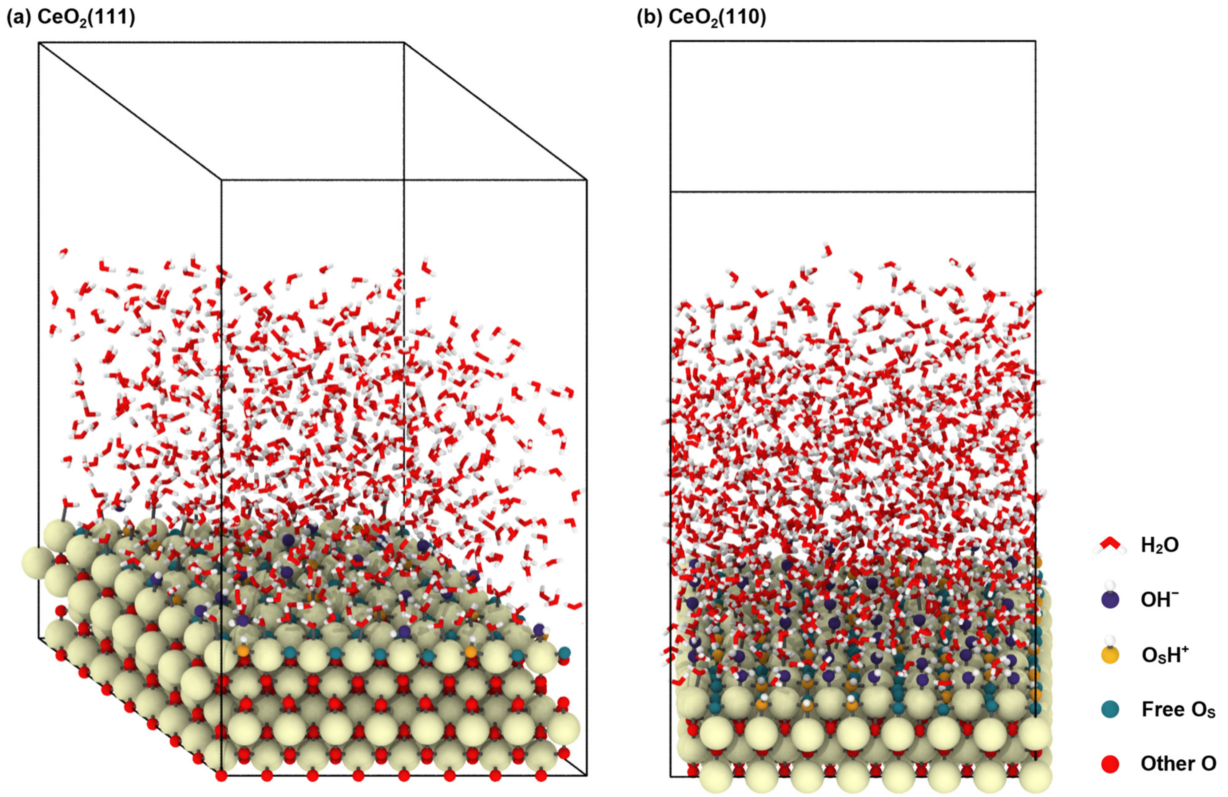

Figure S5. Snapshots of the (a) water/CeO_2(111) and (b) water/CeO_2(110) interface models equilibrated by the NNP-MD simulations.

S.VI. Radial Distribution Functions

The radial distribution functions (RDFs) between the surface Ce atoms and water O atoms calculated from the NNP-MD simulations for $p(8\times8)$ surfaces are shown in Figure S6. The adsorption threshold for each system is also shown. Note that this threshold could affect the values of the proton/hydroxide transfer numbers between surface species.

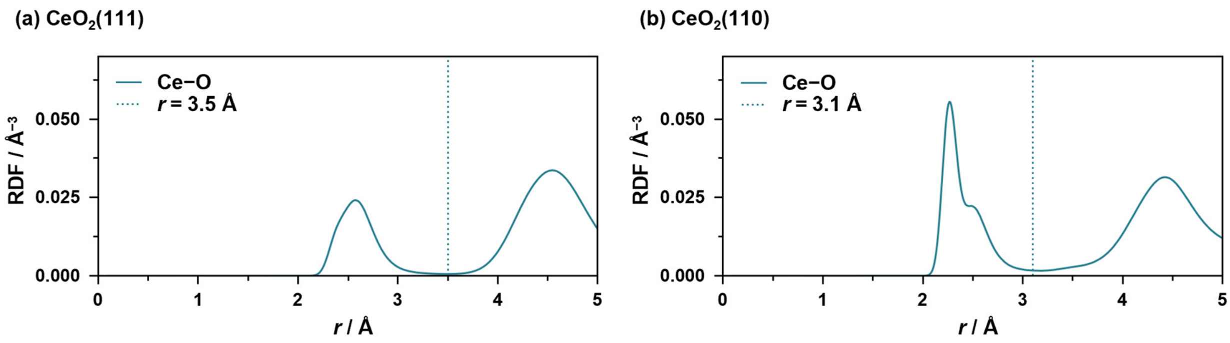

Figure S6. The RDFs of the Ce–O pairs.

S.VII. Decomposed Density Profiles

Figure S7 shows the decomposed profiles of the surface species that are hydrogen bonded to (a–d) surface O atoms or (e) adlayer O atoms. In Figure S7(e), the threshold to determine the bulk region for each system is also given. Figure S8 presents the profiles of the ion species that were not coordinated to the surface Ce atoms, which were generated only transiently during the proton/hydroxide transfer processes. Note that H_3O^+ ions coordinated to the surface Ce atoms were generated quite rarely, and their density profiles are less than 10^{-6} Å^{-3}.

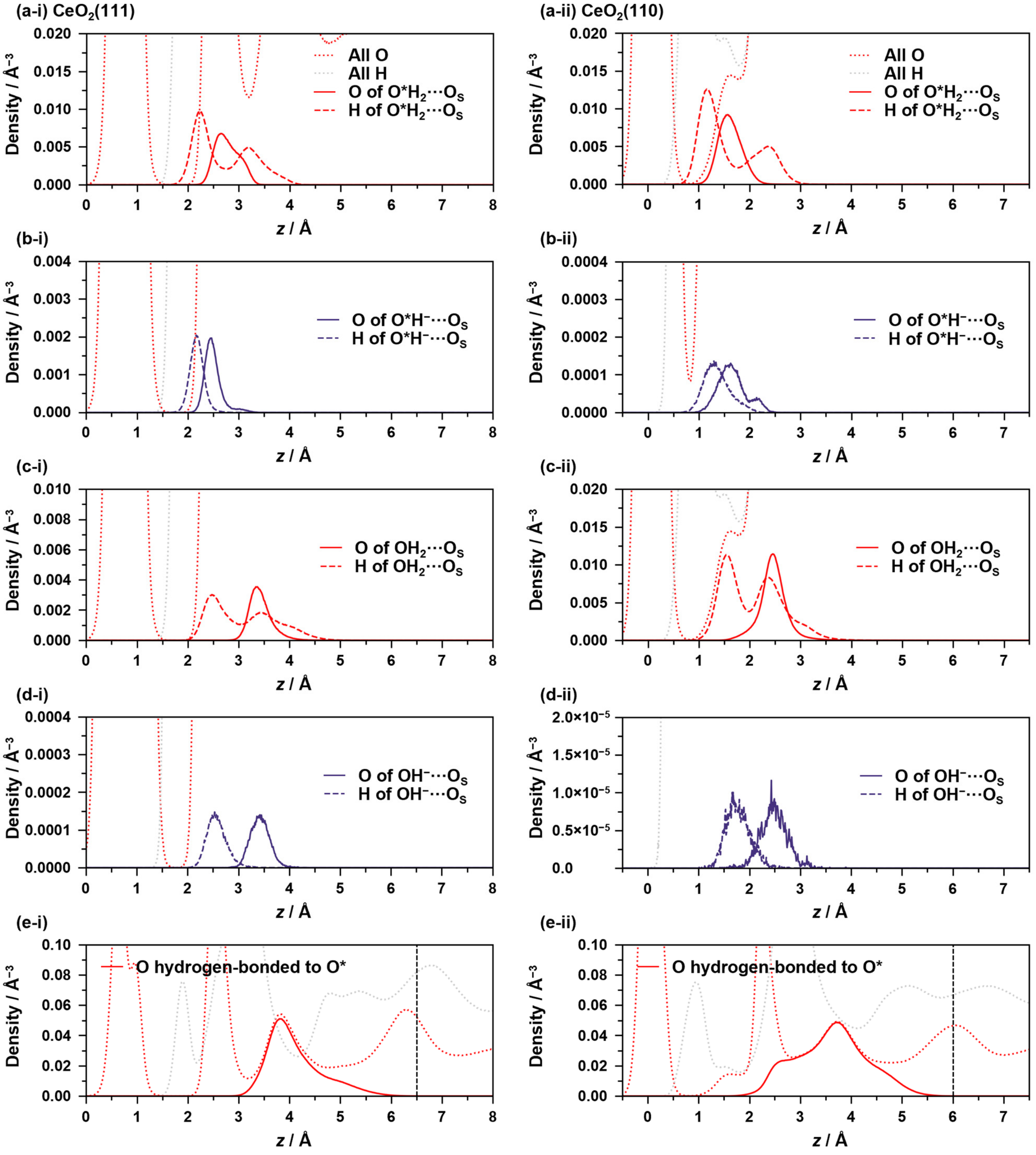

Figure S7. Decomposed density profiles of hydrogen-bonded surface species. Density profiles of total oxygen and hydrogen atoms are also plotted as dotted curves.

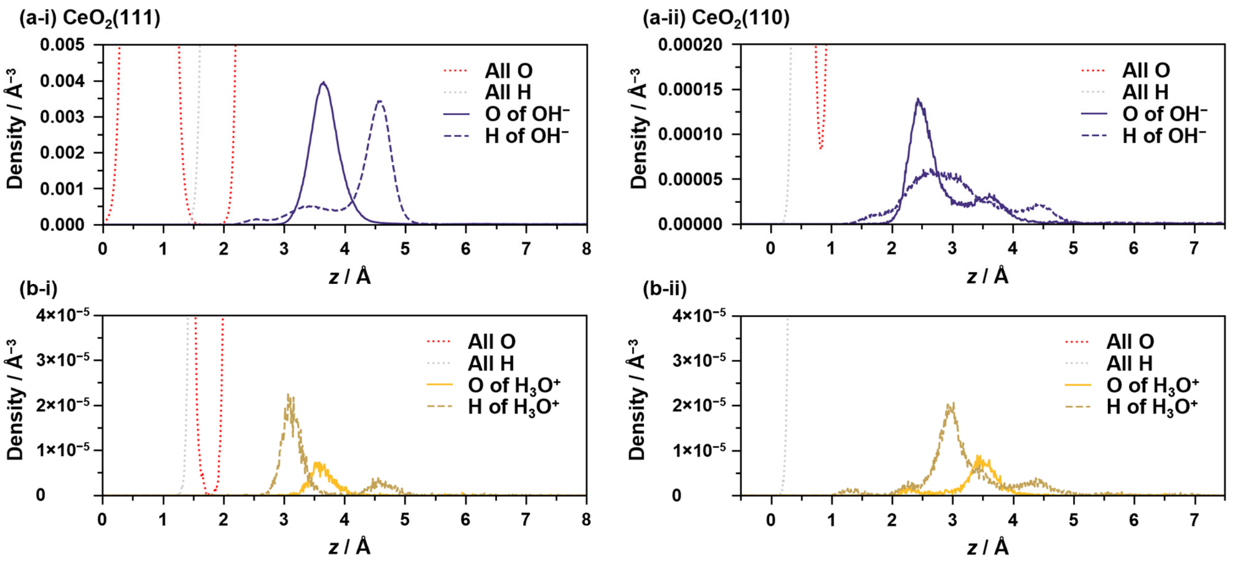

Figure S8. Density profiles of ion species that are not coordinated surface Ce atoms. Density profiles of total oxygen and hydrogen atoms are also plotted as dotted curves.

S.VIII. Supplementary Figures on Proton Hopping Mechanisms

In Figure S9, two supplementary figures regarding the proton transfer mechanisms are given. As depicted in Figure S9(a), even if a proton donor/acceptor adlayer molecule desorbs/adsorbs from a Ce site before/after a hopping event, we treat the molecule as an adlayer molecule. Therefore, the case in Figure S9(a) is classified as an **APT-I** (direct) process rather than an **APT-II** (solvent-assisted) process. Due to this treatment, while two water molecules cannot adsorb on a single Ce site on the $CeO_2(111)$ surface, an **APT-I** process occurs on a single site (Figure S9(b)). Note that, in the case of the $CeO_2(110)$ surface, two water molecules can be coordinated to the same Ce site (see Figure 4(c-ii)).

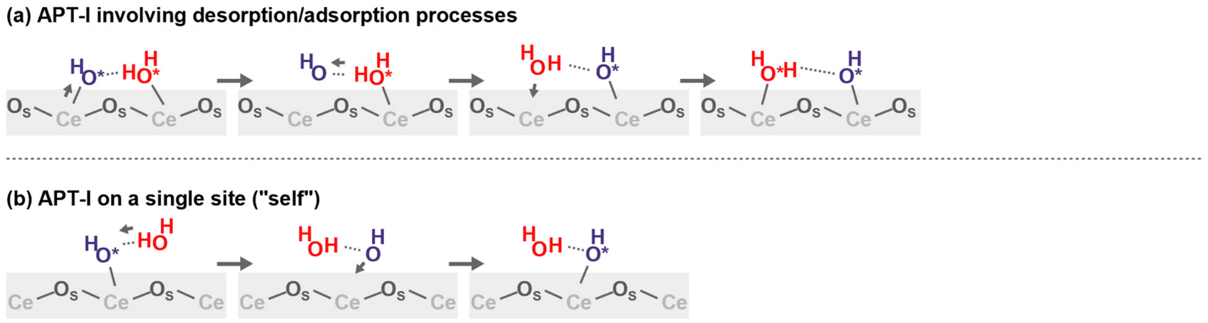

Figure S9. Supplementary figures regarding proton transfer mechanism classifications.

S.IX. Difference Caused by Proton Assignment Procedure

In this section, we introduce the stable state picture (SSP)[9] assignment for comparison with the nearest-neighbor (NNB) assignment of the hydrogen-oxygen bond. While SSP criteria for proton hopping chemical species have already been proposed to analyze population correlation functions,[10] they might be too complicated to apply to a relayed transport analysis, because their strict application to a chain hopping process would require tracking all involved molecules until they are considered to be in stable states. In the following, we propose a simple SSP focusing only on proton states.

Initially at time $t = 0$, all hydrogens are assigned by using the NNB criterion. At time $t > 0$, if a hydrogen atom has only one O atom within 1.1 Å and no other O atoms within 1.5 Å, the hydrogen atom is regarded to be stably bonded to the O atom. These thresholds were determined based on the O−H RDF of bulk water (see Figure S10). If a hydrogen atom does not satisfy this criterion, the hydrogen atom is considered to be still bonded to the previously bonded O atom. By using this SSP assignment, we can eliminate counting rapid recrossing around transition states of proton hopping.

A total of 401,647 and 85,747 proton hopping events were observed for 4 ns equilibration NNP-MD simulations of the water/CeO_2(111) and (110) interfaces, respectively, under the NNB assignment. These numbers were reduced to 72,381 and 8,546 under the SSP assignment. In Table S3 and Table S4, the number of proton/hydroxide ion transfers via the NNB and SSP assignments are shown, omitting event classes that occur only less than twice during 4 ns simulations of $p(8\times8)$ surfaces. Note that each transfer mechanism involves multiple proton hopping events. For reference, the number of transfers that are different from the reverse of the previous transfer (New) and the same as the reverse of the previous transfer (Rattling) are also given. The procedure for identifying "New" and "Rattling" transfers is explained in Section S.XI**.**

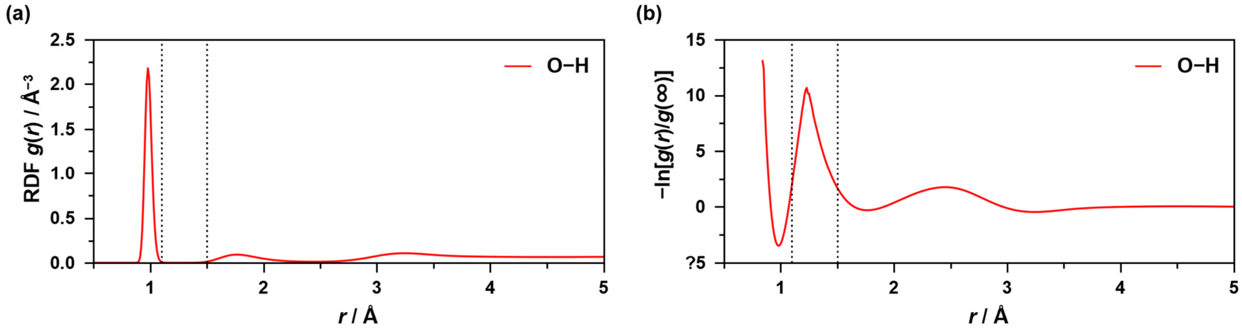

Figure S10. (a) The RDF of the bulk water O−H pair. The bulk water MD trajectory explained in Section 3.5 was used to calculate the RDF. (b) The logarithmic plot of the RDF.

Table S3. Transfer event frequencies calculated from structures sampled every 5 fs from 4 ns NNP-MD trajectories of the water/$CeO_2(111)$ interface. Event classes occurring with a frequency of less than 0.01 ns^{-1} per primitive surface unit cell (approximately twice during 4 ns simulations of $p(8\times8)$ surfaces) are omitted.

$CeO_2(111)$			ns^{-1} primitive surface^{-1}					
			NNB			SSP		
Reaction	Type	Pair	Total	New	Rattling	Total	New	Rattling
SPF	I	nn	25.93	7.27	18.66	6.08	3.02	3.06
	II	nn	1.03	0.66	0.37	0.54	0.36	0.18
		other	0.05	0.05	0.00	0.04	0.04	0.00
	Failed		4.92	–	–	0.49	–	–
SPR	I	nn	25.99	7.34	18.65	6.14	3.34	2.80
	II	nn	0.99	0.77	0.22	0.49	0.41	0.08
		other	0.03	0.03	0.00	0.02	0.02	0.00
APT	I	nn	60.90	14.54	46.36	14.57	6.81	7.86
		self	1.41	–	–	0.77	–	–
	II	nn	128.27	43.84	84.43	62.43	25.00	37.43
		self	0.11	–	–	0.02	–	–
		other	0.13	0.13	0.00	0.14	0.14	0.00
	Failed		517.43	–	–	52.54	–	–
SIR			3.54	–	–	0.08	–	–
AHT		nn	0.04	0.04	0.00	0.05	0.05	0.00

Table S4. Transfer event frequencies calculated from structures sampled every 5 fs from 4 ns NNP-MD trajectories of the water/CeO_2(110) interface.

CeO_2(110)								ns^{-1} primitive surface^{-1}
				NNB			SSP	
Reaction	Type	Pair	Total	New	Rattling	Total	New	Rattling
SPF	I	nn	1.17	0.75	0.42	0.22	0.17	0.05
		nnn	1.81	1.21	0.60	0.38	0.33	0.05
	II	nn	0.35	0.21	0.14	0.22	0.15	0.07
		nnn	1.51	0.78	0.73	0.92	0.55	0.37
	Failed		18.06	–	–	1.55	–	–
SPR	I	nn	1.14	0.10	1.04	0.20	0.07	0.13
		nnn	1.80	0.10	1.70	0.37	0.06	0.31
	II	nn	0.38	0.25	0.13	0.25	0.18	0.07
		nnn	1.52	0.66	0.86	0.93	0.51	0.42
APT	I	*x*	4.34	0.83	3.51	1.08	0.32	0.76
		y	1.40	0.29	1.11	0.38	0.18	0.20
		self	0.44	–	–	0.09	–	–
	II	*x*	0.85	0.46	0.39	0.55	0.34	0.21
		y	5.67	2.04	3.63	3.62	1.48	2.14
		xy	0.38	0.24	0.14	0.25	0.16	0.09
		self	0.03	–	–	0.02	–	–
	Failed		60.21	–	–	2.67	–	–
SPT	I	*x*	2.48	0.44	2.04	1.07	0.32	0.75
	II	*x*	0.22	0.11	0.11	0.16	0.10	0.06
	Failed		0.38	–	–	0.00	–	–
SIR			7.92	–	–	0.15	–	–
AHT		*x*	0.03	0.02	0.01	0.02	0.02	0.00

While the number of transfers labeled “Rattling” significantly reduced under the SSP assignment, the changes in “New” transfers are relatively moderate. This implies that distinguishing “New” transfers from “Rattling” transfers itself causes eliminating recrossing transfers similar to the SSP.

S.X. Histogram of Lifetime of Transient Species during SPF/SPR/SPT-II Processes

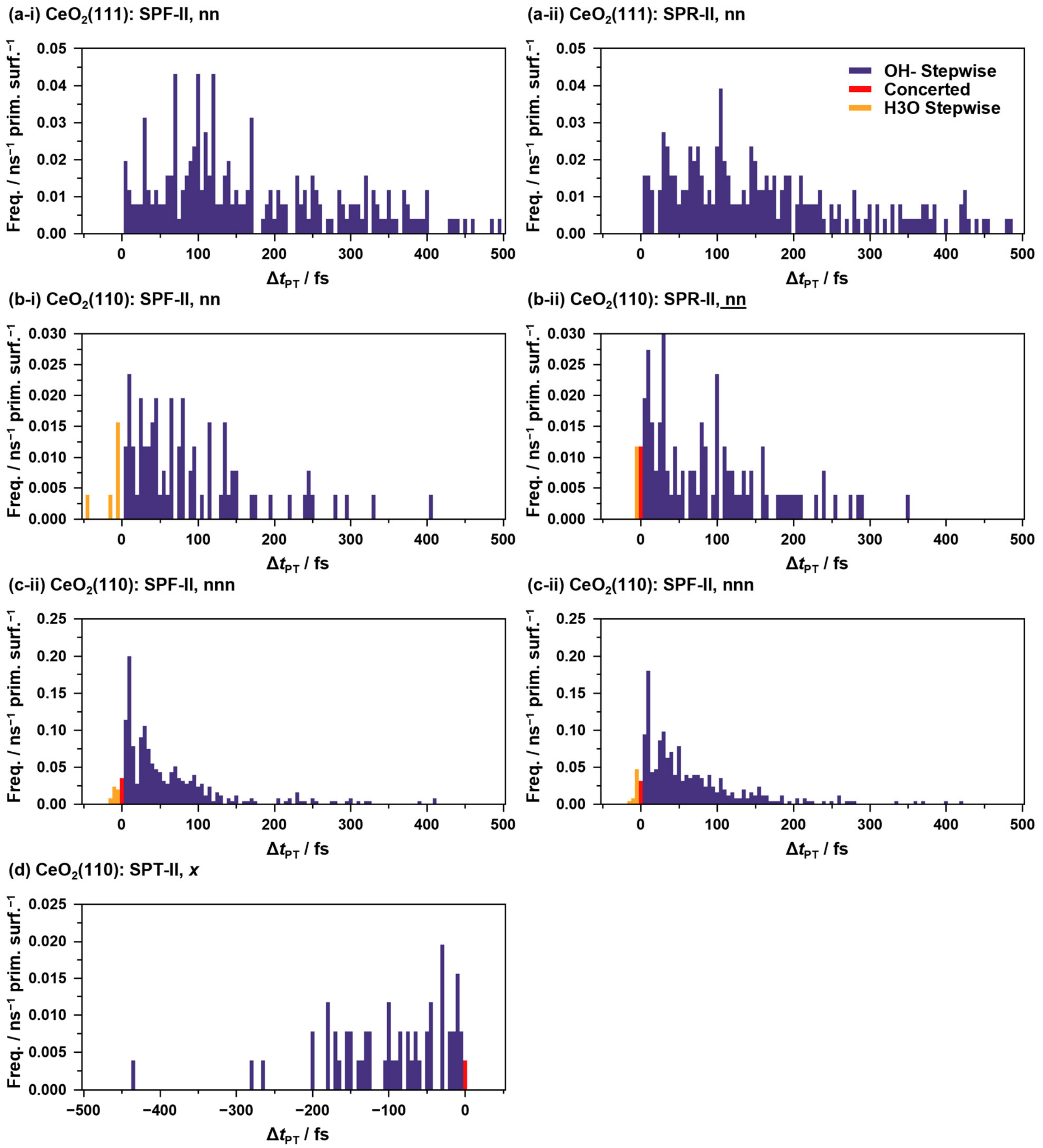

Figure S11. Histograms of $\Delta t_{PT} = t_b - t_a$ during **SPF/SPR/SPT-II** processes. Those during **APT-II** processes are given in Figure 5, and t_a and t_b are defined in Figure 3.

S.XI. Details of Stochastic Models for cMSM/csMSM Analysis

In this section, we describe the details of the cMSM/csMSM we employed. As explained in Section 3.4, our models are described as a set of acid and base lattice sites that have occupied (by adlayer hydroxide ions and protons, respectively) and unoccupied states. In the following, adlayer hydroxide ions are referred to as proton hole states (**PHS**), and proton excess states are abbreviated as **PES**. Each **PHS**/**PES** is assigned a unique ID at its formation , and it moves between the lattice sites. Each site can be multiply occupied.

In order to distinguish "rattling" reactions from others, we introduce quasi-particles, referred to as "trackers" here.

- To track a "rattling" **SPF** reaction that occurs subsequently after an **SPR** reaction on the same acid/base pair sites, we place an **SPF** tracker on an acid/base pair sites when its pair **SPR** reaction occurs on the sites and both of the sites are unoccupied. The transfer mechanism (direct or solvent-assisted) of the **SPR** is also recorded. When an **SPF** reaction occurs on the pair sites that has an **SPF** tracker and its transfer mechanism is the same, the reaction is considered to be a "rattling" reaction. An **SPF** tracker is removed when the occupancy state of either of its pair sites is changed.
- To track a "rattling" **SPR** reaction, we record a **PHS**/**PES** ID pair with its sites and mechanism when an **SPF** reaction occurs as an **SPR** tracker. When an **SPR** reaction occurs on a **PHS**/**PES** pair that has an **SPR** tracker and its transfer mechanism is the same, the reaction is considered "rattling". An **SPR** tracker is deleted when either of its **PHS**/**PES** moves or disappears.
- To track "rattling" transfer, when a **PHS**/**PES** moves and the site it previously occupied is now unoccupied, we record the ID, its mechanism, and the previous site as an **APT**/**AHT**/**SP** tracker. When a transfer reaction occurs to a site that has the corresponding tracker and its transfer mechanism is the same, the reaction is regarded to be "rattling". A transfer tracker is removed when its corresponding **PHS**/**PES** moves or disappears or when another new **PHS**/**PES** is generated at the corresponding site.
- The handling of the **APT-II** trackers on the CeO_2(111) surface is modified to account for "rattling" transfers between acid sites consisting of the tetrahedral structure in Figure 4(c-i) as follows. The **APT-II** trackers are retained as long as the corresponding **PHS** and its **APT-II** trackers form a triangle on the nearest acid lattice sites. If the **PHS** moves to a site that is not part of the previous triangle, the old trackers are deleted.

Considering a space consisting of **PHSs**/**PESs**, sites, and trackers in this way, we can distinguish "rattling/non-rattling" reactions. A "rattling" interval is calculated by using the generation time of the corresponding tracker.

In the actual cMSM/csMSM analysis, the rates corresponding to the escape/adsorption of **PHSs**/**PESs** from the surface region were also measured. However, since KMC simulations with/without the rates agreed well in the reproducibility of the coverage/**PHS** lifetime/diffusion, we adopted the results of the KMC simulation without the rates to simplify the picture.

In the cMSM/csMSM in Section 3.4, we assume that the reaction rates of **SPF**/**SPR**/**SPT** depend on the number of **PES** around the corresponding base sites and that the reaction rates of **SPF**/**SPR**/**APT**/**AHT** depend on the

number of **PHS** occupancies of the corresponding acid sites. To examine this environment dependence of the reaction rates, we show the results of two different models. In the first model (csMSM-a), we omit all environment dependencies while maintaining the semi-Markov nature of the model. In the second model (csMSM-b), we consider not only the number of PES around the base sites but also their spatial configurations to the reaction site pairs. Thus, the csMSM-a and csMSM-b are simpler and more complicated variants of the csMSM, respectively.

Figure S12 shows the results of the KMC based on the reaction rates calculated by using the csMSM-a and csMSM-b, including the same results in Figure 6. The KMC based on the csMSM-a does not reproduce the hydroxylation ratio of the surface O sites, and there is no significant improvement in the KMC based on the csMSM-b compared to that of the csMSM. Therefore, we concluded that the csMSM contained sufficient environment dependence in this regard.

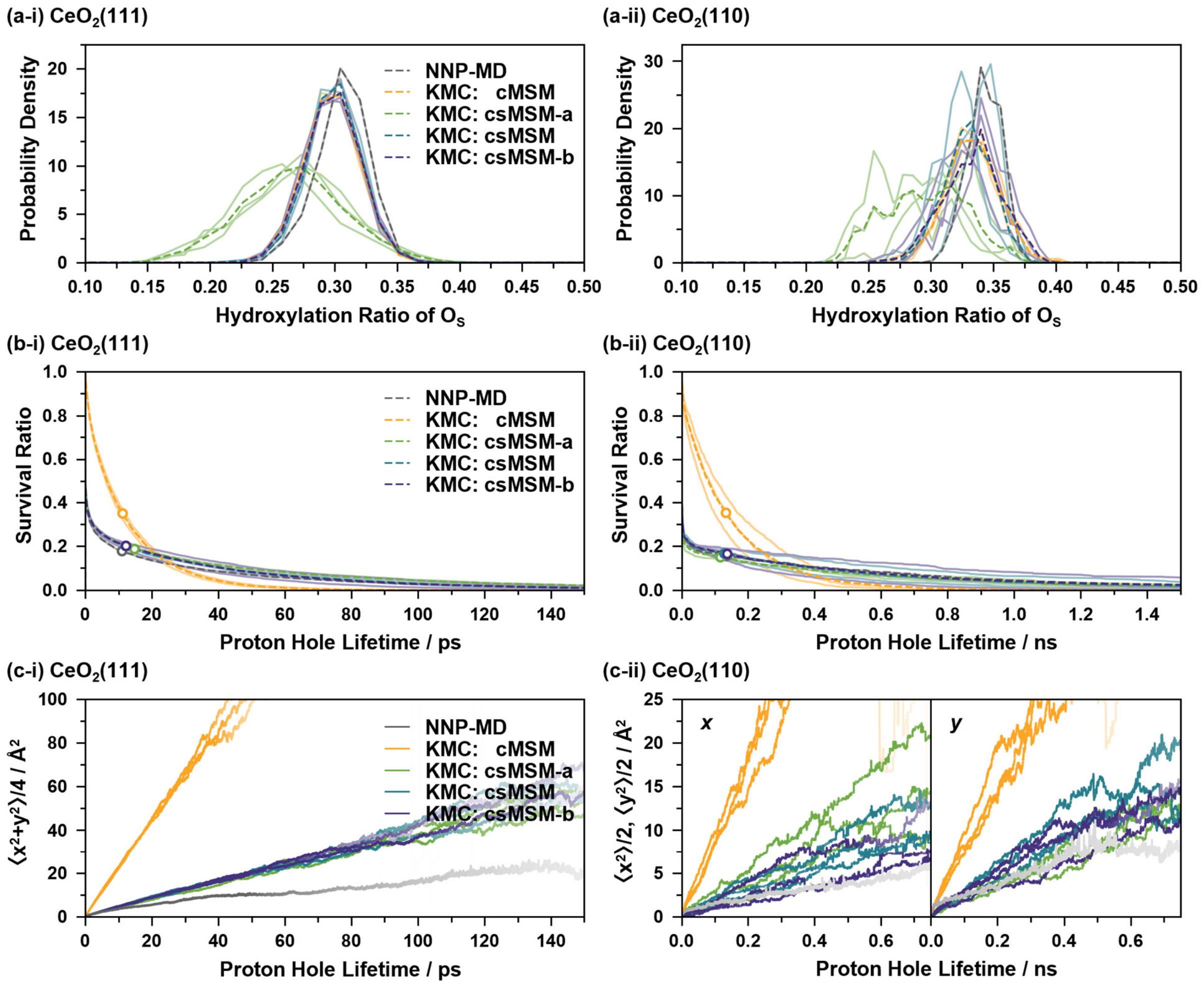

Figure S12. (a) Probability density distributions of the hydroxylation ratio of the surface O atoms, (b) proton hole survival functions, and (c) mean square displacements (MSD) of the proton holes.

S.XII. Parameters in KMC Simulations

In this study, we conducted three independent KMC simulations for each model. For the “non-rattling” part, we used (1) average, (2) upper, and (3) lower bounds of the 95% Clopper–Pearson confidence intervals of the reaction rates. For the “rattling” part, we used only the average values of the time-dependent reaction rates. The initial lattice states of the KMC simulations were taken from those of the NNP-MD equilibrium trajectories.

S.XIII. Environment Dependent Reaction Rates

In this section, we show a part of the raw results of the csMSM analysis. In our cMSM/csMSM models, the reaction rates of reactions involving metal acid sites depend on the number of assigned OH^- ions to the sites (Number of hole occupations; **#PHO**), and those of reactions involving surface oxygen base sites depend on the number of protonated base sites around the sites (Number of surface oxygen protonation, **#SOP**). In Table S5 and Table S6, the estimated reaction rates are shown, while those of the transfers observed only less than twice during 4 ns simulations of $p(8\times8)$ surfaces are omitted. Here, "src." and "dst." represent source and destination of transfers. The values of Figure 6(c) are the averages of raw reaction rates weighted by their frequency of reaction opportunity.

Table S5. Reaction rates of "non-rattling" reactions based on the csMSM for the water/CeO_2(111) interface.

Reaction	Type	Pair	#PHO of acid site	#SOP around base site	Rate / ns^{-1} $pair^{-1}$ Lower 95%	Average	Upper 95%
SPF	I	nn	0	0	21.74	25.52	29.77
				1	10.32	11.27	12.28
				2	2.94	3.23	3.54
				3	0.68	0.81	0.96
			1	0	26.88	37.98	52.13
				1	2.81	3.85	5.15
				2	0.41	0.62	0.89
				3	0.03	0.08	0.17
	II	nn	0	0	5.93	7.77	10.00
				1	0.96	1.25	1.59
				2	0.05	0.09	0.15
				3	0.00	0.02	0.05
			1	0	1.62	5.00	11.66
SPR	I	nn	1	0	14.58	15.80	17.10
				1	28.27	30.81	33.51
				2	45.16	52.95	61.71
				3	22.44	49.07	93.13
			2	0	105.30	188.11	310.17
				1	111.35	191.12	305.91
				2	117.28	291.61	600.36
	II	nn	1	0	1.86	2.29	2.79
				1	2.26	2.95	3.77
				2	2.89	4.80	7.49

Reaction	Type	Pair	#PHO of src. acid site	#PHO of dst. acid site	Rate / ns^{-1} $pair^{-1}$ Lower 95%	Rate	Upper 95%
APT	I	nn	1	0	8.55	8.89	9.24
				1	0.41	0.52	0.65
			2	0	16.97	26.77	40.16
	II	nn	1	0	34.19	34.93	35.69
				1	1.53	1.75	1.98
			2	0	140.32	168.69	201.12
				1	5.79	17.84	41.64
AHT		nn	1	0	0.01	0.03	0.06

Table S6. Reaction rates of "non-rattling" reactions based on the csMSM for the water/CeO_2(110) interface.

Reaction	Type	Pair	#PHO of acid site	#SOP around base site (x, y)	Rate / ns^{-1} $pair^{-1}$ Lower 95%	Average	Upper 95%
SPF	I	nn	0	0, 0	0.11	0.22	0.40
				0, 1	0.14	0.26	0.42
				0, 2	0.26	0.57	1.08
			1	0, 0	0.02	0.10	0.28
				0, 1	0.40	0.54	0.72
				0, 2	0.61	0.78	0.99
				1, 1	0.54	0.84	1.24
			2	0, 1	0.02	0.05	0.11
		nnn	0	0, 0	1.16	1.47	1.83
				0, 1	0.31	0.48	0.72
				0, 2	0.09	0.31	0.80
			1	0, 0	1.17	1.43	1.72
				0, 1	0.71	0.89	1.10
				1, 1	0.02	0.05	0.13
	II	nn	0	0, 0	0.12	0.24	0.42
				0, 1	0.31	0.47	0.68
				0, 2	0.10	0.32	0.74
			1	0, 0	0.03	0.07	0.15
		nnn	0	0, 0	1.08	1.37	1.71
				0, 1	1.36	1.71	2.12
				0, 2	0.61	1.11	1.86

			1	0, 0	0.04	0.09	0.19
				0, 1	0.04	0.08	0.17
				0, 2	0.03	0.12	0.32
SPR	I	nn	1	0, 0	0.03	0.08	0.15
				0, 2	0.04	0.21	0.60
			2	0, 0	0.35	1.06	2.48
				0, 1	0.18	0.89	2.59
		nnn	1	0, 0	0.02	0.06	0.13
				0, 1	0.03	0.06	0.12
			2	0, 0	0.64	3.10	9.06
				0, 1	0.21	0.77	1.98
	II	nn	1	0.09	0.16	0.25	0.09
				0.28	0.41	0.57	0.28
				0.29	0.63	1.20	0.29
			2	0.26	0.95	2.44	0.26
		nnn	1	0.45	0.59	0.75	0.45
				0.54	0.68	0.84	0.54
				0.15	0.33	0.62	0.15
			2	1.03	3.77	9.66	1.03
				0.21	0.77	1.97	0.21
				0.23	0.83	2.12	0.23

Reaction	Type	Pair	#PHO of src. acid site	#PHO of dst. acid site	Rate / ns^{-1} $pair^{-1}$		
					Lower 95%	Rate	Upper 95%
APT	I	*x*	1	0	0.53	0.67	0.84
				1	0.45	0.55	0.65
			2	0	0.38	0.95	1.95
		y	1	0	0.64	0.83	1.05
				1	0.00	0.02	0.04
	II	*x*	1	0	0.71	0.87	1.06
				1	0.03	0.06	0.10
			2	0	0.08	0.40	1.18
		y	1	0	6.71	7.41	8.17
				1	0.18	0.24	0.31
			2	0	10.89	16.39	23.69
				1	0.85	1.49	2.42
		xy	1	0	0.12	0.17	0.23

				1	0.02	0.04	0.06
			2	0	0.17	0.45	0.98
AHT		*x*	2	1	0.11	0.54	1.59

Reaction	Type	Pair	#SOP around src. base site	#SOP around dst. base site	Rate / ns^{-1} $pair^{-1}$ Lower 95%	Rate	Upper 95%
SPT	I	*x*	0, 0	1, 0	0.67	1.27	2.17
				1, 1	0.48	0.83	1.32
				1, 2	0.68	1.21	2.00
			0, 1	1, 0	0.94	1.47	2.19
				1, 1	0.71	1.08	1.57
			0, 2	1, 0	0.58	1.15	2.06
	II	*x*	0, 0	1, 1	0.09	0.22	0.45
			0, 1	1, 0	0.03	0.11	0.28
				1, 1	0.16	0.32	0.55

As seen in Section S.XI, these environment dependencies are essential to describe surface hydroxylation. Mostly, the observed “lateral interaction” was repulsive. For pair formation reactions, denser environments caused lower reaction rates, and the opposite tendency existed for pair recombination reactions. For transfer reactions, if the source site of the proton hole/excess proton was in a dense environment, the interaction promoted the transfer, and *vice versa* for the transfer destination site. An exception is the **SPF** reaction of the “nn” pair for the water/CeO_2(110) interface. Because a formed OH^- could be stabilized by near O_SH^+, a denser environment appeared to promote pair formation reactions.

S.XIV. Rattling Reaction Rates

Some of the "rattling" part of the reaction rates based on the csMSM are given in Figure S13 and Figure S14. Although some of the 50% confidence intervals exceed the plot range, they are plotted as they are to show the relative magnitudes between them.

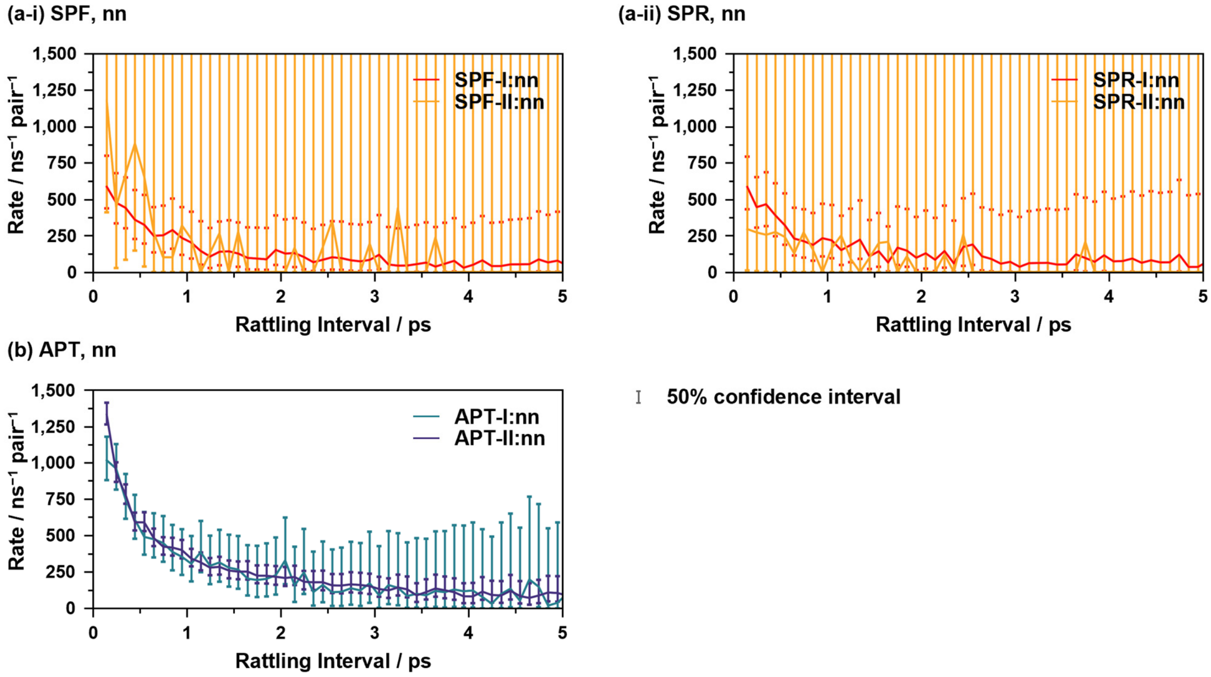

Figure S13. Time-dependent reaction rates of "rattling" reactions for the water/CeO_2(111) interface. The values are averaged every 100 fs, and environment dependences are also averaged.

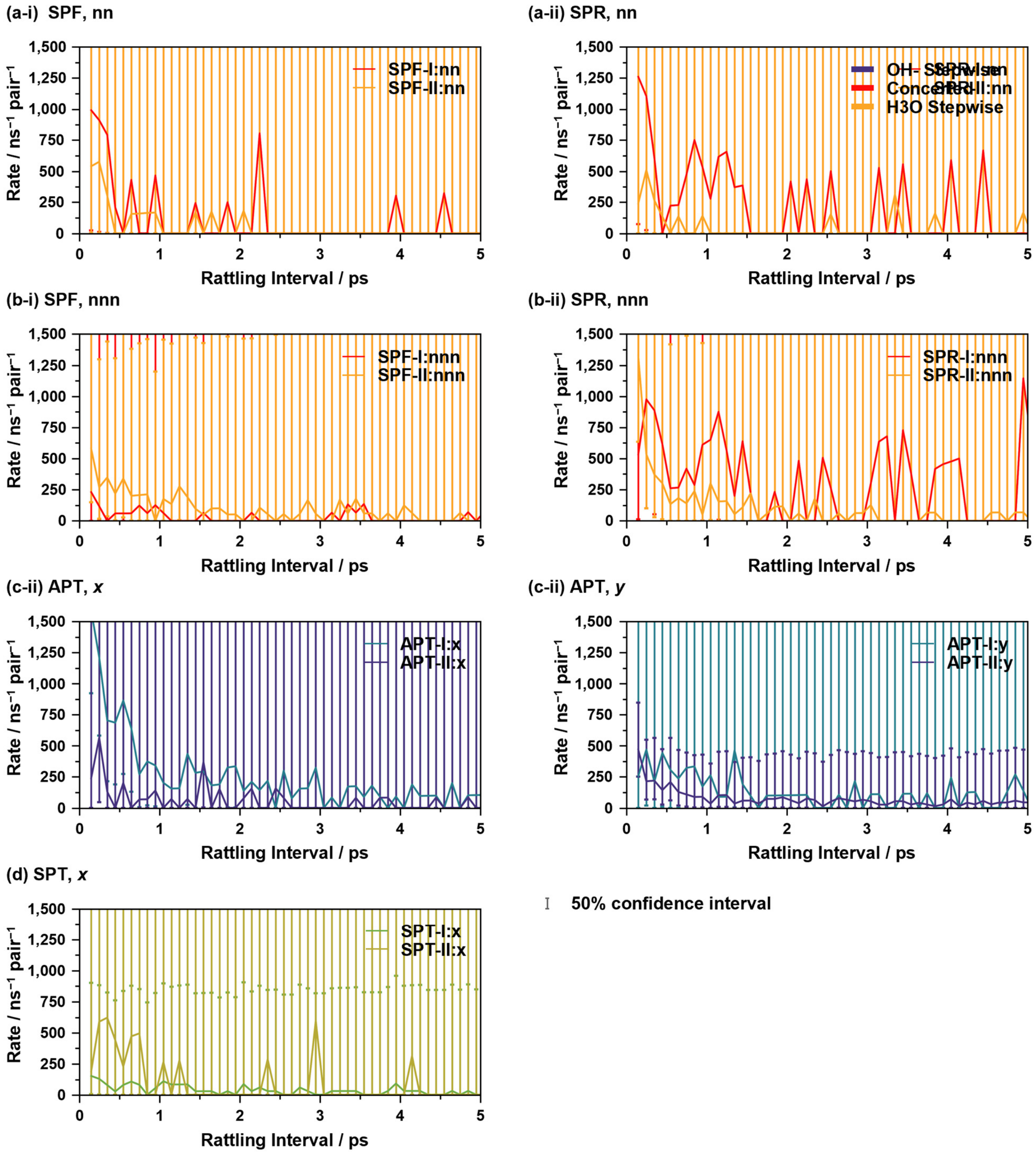

Figure S14. The same plots as in Figure S13, but for reactions for the water/CeO_2(110) interface.

S.XV. Vibrations of Adsorbed Species

Figure S15 shows the OH stretching and H_2O bending motions of adlayer species in order to compare with transient vibrations in Figure 9. We performed at least 1 ns NNP-MD simulations with $p(4\times4)$ slab sizes for the water/CeO_2(111) and water/CeO_2(110) interfaces to increase the sampling resolution to 0.5 fs. For comparison, we also performed at least 1 ns bulk water NNP-MD simulation with 200 water molecules. The motions of the adlayer water molecules were evaluated by sampling the motion of the H_2O* molecules continuously adsorbed on a surface Ce atom for at least 100 fs.

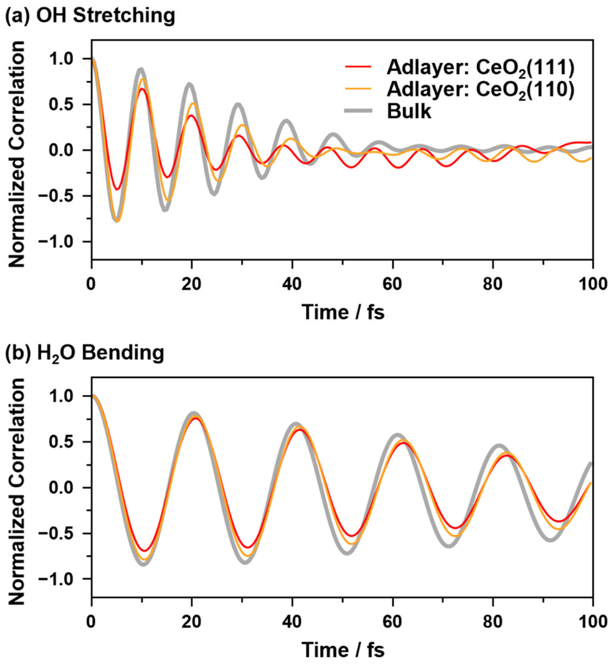

Figure S15. Vibration correlation functions of the adlayer and bulk water (a) OH stretching and (b) H_2O bending.

S.XVI. Combination between SSP Assignment and cMSM/csMSM

To investigate the effects of the proton assignment procedure to the reaction rates, we conducted cMSM/csMSM analysis by using the SSP assignment defined in Section S.IX. The results of the KMC simulations for validation are given in Figure S16, and some of the reaction rates based on the csMSM are shown Figure S17.

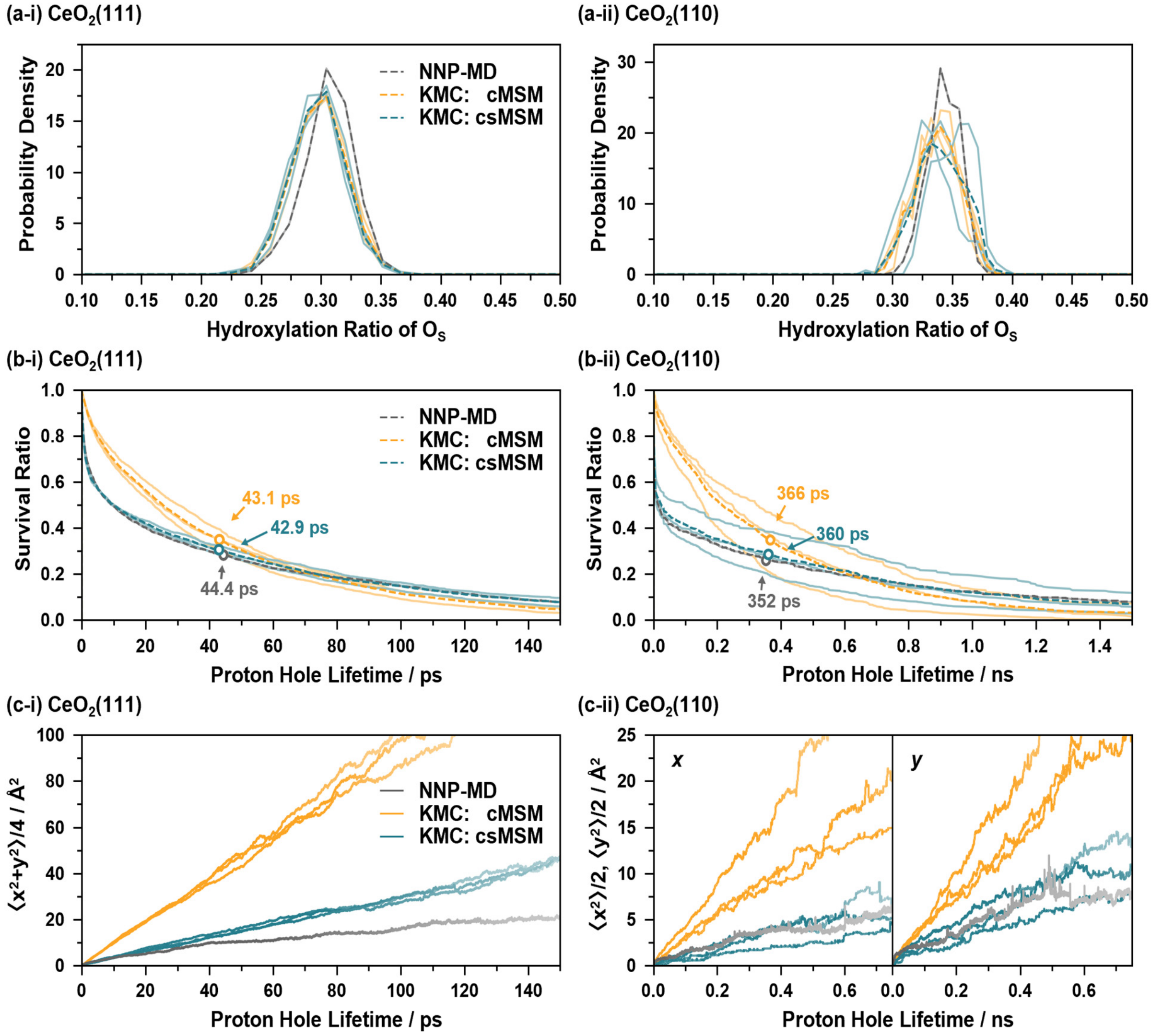

Figure S16. (a) Probability density distributions of the hydroxylation ratio of the surface O atoms, (b) proton hole survival functions, and (c) mean square displacements of the proton holes, obtained based on the SSP assignment. In the case of (c), each curve is plotted thinner as the corresponding survival function becomes less than 20%.

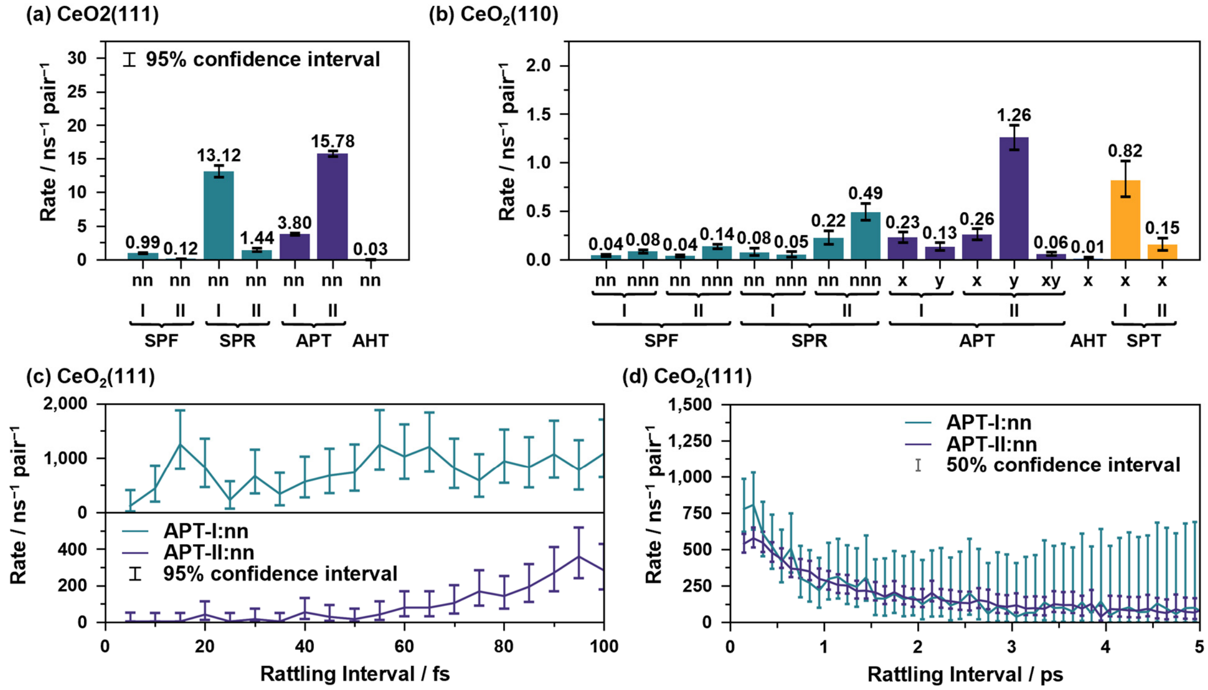

Figure S17. The same plots as in Figure 7 but for values obtained by using the SSP assignment.

As seen in Figure S16, the hole survival functions significantly changed because holes with short lifetimes caused by rapid recrossing were eliminated by the SSP assignment. However, surface hydroxylation ratios and long-range diffusion properties are similar to those of the NNB assignment. Also, while the short-time behavior of the time-dependent reaction rates was significantly affected, as in Figure S17(c), the long-time behavior and constant terms are relatively similar, as in Figure S17(a), (b), and (d). Therefore, the global tendency of the reaction rate we obtained can be considered essential regardless of the details of the proton assignment.

References

(1) Kumar, S.; Rosenberg, J. M.; Bouzida, D.; Swendsen, R. H.; Kollman, P. A. THE Weighted Histogram Analysis Method for Free-Energy Calculations on Biomolecules. I. The Method. *J. Comput. Chem.* **1992**, *13*, 1011–1021.

(2) Tribello, G. A.; Bonomi, M.; Branduardi, D.; Camilloni, C.; Bussi, G. PLUMED 2: New Feathers for an Old Bird. *Comput. Phys. Commun.* **2014**, *185*, 604–613.

(3) Maragliano, L.; Vanden-Eijnden, E. A Temperature Accelerated Method for Sampling Free Energy and Determining Reaction Pathways in Rare Events Simulations. *Chem. Phys. Lett.* **2006**, *426*, 168–175.

(4) Abrams, J. B.; Tuckerman, M. E. Efficient and Direct Generation of Multidimensional Free Energy Surfaces via Adiabatic Dynamics without Coordinate Transformations. *J. Phys. Chem. B* **2008**, *112*, 15742–15757.

(5) Ren, Z.; Liu, N.; Chen, B.; Li, J.; Mei, D. Theoretical Investigation of the Structural Stabilities of Ceria Surfaces and Supported Metal Nanocluster in Vapor and Aqueous Phases. *J. Phys. Chem. C* **2018**, *122*, 4828–4840.

(6) Fernández-Torre, D.; Kośmider, K.; Carrasco, J.; Ganduglia-Pirovano, M. V.; Pérez, R. Insight into the Adsorption of Water on the Clean CeO_2(111) Surface with van Der Waals and Hybrid Density Functionals. *J. Phys. Chem. C* **2012**, *116*, 13584–13593.

(7) Brugnoli, L.; Menziani, M. C.; Urata, S.; Pedone, A. Development and Application of a ReaxFF Reactive Force Field for Cerium Oxide/Water Interfaces. *J. Phys. Chem. A* **2021**, *125*, 5693–5708.

(8) Molinari, M.; Parker, S. C.; Sayle, D. C.; Islam, M. S. Water Adsorption and Its Effect on the Stability of Low Index Stoichiometric and Reduced Surfaces of Ceria. *J. Phys. Chem. C* **2012**, *116*, 7073–7082.

(9) Laage, D.; Hynes, J. T. On the Residence Time for Water in a Solute Hydration Shell: Application to Aqueous Halide Solutions. *J. Phys. Chem. B* **2008**, *112*, 7697–7701.

(10) Quaranta, V.; Behler, J.; Hellström, M. Structure and Dynamics of the Liquid–Water/Zinc-Oxide Interface from Machine Learning Potential Simulations. *J. Phys. Chem. C* **2019**, *123*, 1293–1304.
